# Manufacturing, Microstructure, and Mechanics of 316L SS Biomaterials by Laser Powder Bed Fusion

**DOI:** 10.3390/jfb16080280

**Published:** 2025-07-31

**Authors:** Zhizhou Zhang, Paul Mativenga, Shi-Qing Huang

**Affiliations:** 1 School of Mechanics and Construction Engineering, Jinan University, Guangzhou 510632, China; 2 Laser Processing Research Laboratory, School of Engineering, The University of Manchester, Manchester M13 9PL, UK; 3 College of Packaging Engineering, Jinan University, Zhuhai 519070, China

**Keywords:** laser powder bed fusion, 316L stainless steel, mechanical properties, additive manufacturing, metallography, biomaterials

## Abstract

Laser powder bed fusion (LPBF) is an advanced additive manufacturing technology that is gaining increasing interest for biomedical implants because it can produce dense, patient-specific metallic components with controlled microstructures. This study investigated the LPBF fabrication of 316L stainless steel, which is widely used in orthopedic and dental implants, and examined the effects of laser power and scanning speed on the microstructure and mechanical properties relevant to biomedical applications. The study achieved 99.97% density and refined columnar and cellular austenitic grains, with optimized molten pool morphology. The optimal LPBF parameters, 190 W laser power and 700 mm/s, produced a tensile strength of 762.83 MPa and hardness of 253.07 HV_0.2_, which exceeded the values of conventional cast 316L stainless steel. These results demonstrated the potential of optimized LPBF 316L stainless steel for functional biomedical applications that require high mechanical integrity and biocompatibility.

## 1. Introduction

Metallic biomaterials are indispensable in the field of orthopedic and dental implants, where mechanical strength, ductility, and structural reliability are essential for clinical use [1,2,3]. Among these materials, 316L austenitic stainless steel is widely employed due to its high strength-to-weight ratio, excellent toughness, weldability, and established biocompatibility in load-bearing applications [4,5,6]. While traditional manufacturing methods such as casting and forging have historically dominated implant fabrication, these approaches are limited in their ability to produce complex geometries and patient-specific devices required by modern medicine [7,8,9,10].

Additive manufacturing (AM), especially laser powder bed fusion (LPBF), has rapidly emerged as a transformative technology that overcomes these limitations, enabling the creation of intricate, dense, and customized metallic parts layer by layer directly from digital models [11,12,13]. LPBF offers unique advantages for biomedical applications, including the ability to design implants with optimized internal architectures for load transfer and osseointegration, as well as to tailor microstructure through precise control of processing parameters [8,14,15].

However, LPBF of 316L stainless steel is associated with unique microstructural features and defect populations compared to conventionally processed materials [16]. Rapid solidification during LPBF leads to the formation of hierarchical structures, including cellular and columnar austenite grains [13], pronounced texture [17], and in some cases, residual porosity or microcracking [18], which can critically influence mechanical performance. The success of implants extends beyond simple composition; it is closely linked to the phase constitution, microstructure, and mechanical properties that ultimately dictate clinical performance, including resistance to fracture, corrosion, and implant failure [19,20].

A critical factor influencing the functional reliability of implants is the control of phase structure and grain morphology. The predominance of the austenitic phase in 316L stainless steel imparts desirable mechanical properties, such as high ductility and work-hardening capacity, while also minimizing the risk of stress-induced martensitic transformation that could degrade biocompatibility or induce corrosion [21]. Fine-grained cellular or columnar austenitic structures, often developed through rapid solidification in advanced manufacturing processes like laser powder bed fusion (LPBF), enhance both strength and toughness, properties essential for implants exposed to repetitive physiological loading [22]. Moreover, a homogeneous and stable austenitic matrix helps to suppress localized corrosion and pitting, thereby extending implant longevity and reducing the risk of adverse tissue reactions [23]. Biological responses at the tissue–implant interface are profoundly affected by the phase purity and surface microstructure of the implant. Studies have shown that controlled cellular and columnar austenite, with minimized secondary phases and refined grain boundaries, can improve protein adsorption, promote osteoblast adhesion, and enhance osseointegration, all of which are prerequisites for long-term implant fixation and stability [24]. Conversely, the presence of ferritic or martensitic phases, excessive porosity, or elemental segregation may trigger local inflammatory responses or fibrous encapsulation, undermining clinical outcomes [25].

Other factors have a great impact on biomaterial qualities, such as scanning mode, laser power, scanning speed, scanning spacing, layer thickness, and scanning strategy [26]. Li et al. [27] studied the scanning trajectory characteristics, densification, and tensile properties of LPBF samples made of 316L stainless steel powder at different scanning speeds. The results showed that when the laser power was 100 W and the scanning speed ranged from 90 to 180 mm/s, the porosity increased and the tensile strength decreased with the increase in scanning speed. Wang et al. [28] obtained LPBF-formed 316L samples with a relative density of 99.99% under the conditions of 150 μm layer thickness, process parameters of 380 W and 333 mm/s (the construction speed was 3 to 10 times faster than previous studies). As the scanning speed decreased, the grain size increased and the tensile strength decreased. Suryawanshi et al. [29] used strip and chessboard laser scanning strategies to manufacture LPBF-formed 316L specimens and studied the effects of scanning strategies on tensile strength, fracture toughness, and fatigue crack growth. They found that under the chessboard scanning strategy, the tensile strength of the specimen reached a maximum of 621.7 MPa, the fracture toughness was 79.6 MPa√m, and the periodic crack growth rate was the lowest at 7.8 MP m0.5. Li et al. [30] investigated the effects of laser power, scanning speed, and layer thickness on the densification of 316L stainless steel.

Nevertheless, despite promising progress, the underlying mechanisms linking LPBF parameters, microstructural evolution, and the resulting mechanical performance remain incompletely understood [31,32,33]. There remains a pressing need for systematic studies that elucidate these relationships, especially under processing windows directly relevant to clinical-grade implant fabrication. In particular, the interplay between laser power, scanning speed, and the development of grain morphologies and defect structures requires further elucidation to inform process optimization and material selection [34,35,36].

In this context, the present study systematically investigates the effects of key LPBF process parameters—specifically laser power and scanning speed—on the microstructure and the mechanical properties most relevant to biomedical implants, namely tensile strength and Vickers hardness, of 316L stainless steel. By evaluating tensile strength and hardness with controlled processing variables, this work aims to identify optimal LPBF parameter windows to manufacture high-integrity, bio-compatible metallic implants, thereby advancing the understanding and application of additive manufacturing in the biomedical field.

## 2. Methodology

The LPBF equipment used in this study is laser powder bed fusion equipment model FS121M (FARSOON TECHNOLOGIES Co., Ltd., Changsha, China). During the process, a continuous flow of Argon gas circulates within the build chamber. This prevents the metal powder from reacting with oxygen at high temperatures, which could lead to explosions, and it removes spatter produced by the instant plasma formation of 316L stainless steel powder under the laser [31].

As shown in Figure S1, the main equipment’s control system is operated via an embedded computer, which runs the slicing software BuildStar^TM^ 1.6 and process control software, MakeStar^TM^ 2.6.1 (FARSOON TECHNOLOGIES Co., Ltd., Changsha, China). The top section of the main equipment contains a safety door with a glass shield for observing the workspace environment. The machine’s key auxiliary equipment is a gas circulation system connected to the main equipment’s intake and exhaust ducts. The exhaust duct removes spatter from the build chamber, while the purified protective gas is recirculated back into the chamber through the intake duct, ensuring efficient reuse of the shielding gas.

### 2.1. LPBF Processing Parameters

During the printing process, the laser spot size significantly affected the quality and efficiency of the fabricated components. Periodic adjustments to the spot size were necessary to maintain optimal performance. As shown in Figure S2, the laser spot calibration process involved melting tracks on an aluminum plate, where the width of the white lines corresponded to the diameter of the laser spot. Smaller laser spots increased the energy density delivered to the powder, which could lead to spattering and balling [37]. In contrast, an appropriately sized laser spot improved the part precision, increased the density, and enhanced the mechanical properties. If the laser spot was too large, the reduced energy density resulted in incomplete powder melting and weak bonding between adjacent melt pools. This condition led to interlayer cracks that severely compromised the mechanical properties of the printed parts. Based on these considerations, the laser spot size for fabricating 316L stainless steel specimens in this study was set to 138 µm.

In metal 3D printing, parts were built layer by layer. To reduce stress concentrations that could cause warping and deformation, it was important to adjust the orientation between successive layers. Setting a layer rotation angle of 67° minimized overlap between layers during the LPBF process, which improved both the density and mechanical properties of the printed parts [38]. The scanning strategy required changing the laser beam orientation between adjacent layers to promote better adhesion and enhance mechanical performance. For example, when the first layer was scanned at 0°, the subsequent layer was scanned at a different angle. This alternating approach strengthened interlayer bonding. In this study, a unidirectional scanning strategy was used for metallographic specimens, with layer rotation angles of 0° and 67°, respectively.

It is important to note that the energy density *E* (J/mm^3^) better represents the energy input for LPBF specimens compared to other parameters. It is calculated using the following formula:

*E* = *P*/(*v* × *h* × *t*) (1)

Here, *P* represents laser power (W), *v* is the scanning speed (mm/s), *h* is the hatch spacing (mm), and *t* is the layer thickness (mm). Laser energy density is determined by the initial LPBF process conditions, including laser power, scanning speed, hatch spacing, and layer thickness. In this study, a parametric investigation was conducted to explore the effect of laser energy density on the densification and microstructural morphology of LPBF-fabricated SS316L parts. By adjusting the laser scanning speed (*v*) and laser power (*P*), variations in energy density were controlled. The hardness and tensile properties of the fabricated specimens were tested to identify an optimal LPBF process window for producing high-quality 316L stainless steel components.

Laser power and scanning speed were selected as the primary process parameters to investigate their effects on the mechanical properties of 316L stainless steel fabricated by LPBF. Tensile test specimens were prepared on a 45 steel substrate using a laser power range of 140–190 W and scanning speeds from 600 to 900 mm/s. Other processing parameters were kept constant, following the manufacturer’s recommendations: laser spot diameter of 138 µm, hatch spacing of 90 µm, and powder layer thickness of 30 µm.

To suppress the epitaxial growth of columnar grains and increase the degree of grain orientation disorder within the melt pool, an interlayer rotation angle of 67° was applied [39]. Alsalla et al. [40] found that flat specimen orientations provided superior mechanical properties; accordingly, this study adopted the same structural orientation. To prevent oxidation and powder spattering during the LPBF process, the oxygen level was maintained below 5000 ppm, and the entire build was carried out in a nitrogen-filled protective chamber with a circulating gas pressure below 100 Pa.

The specific processing parameters are summarized in Table 1, where P is the laser power (W), v is the scanning speed (mm/s), h is the hatch spacing (mm), t is the powder thickness (mm), and E is the energy density (J/mm^3^).

**Figure 1:**
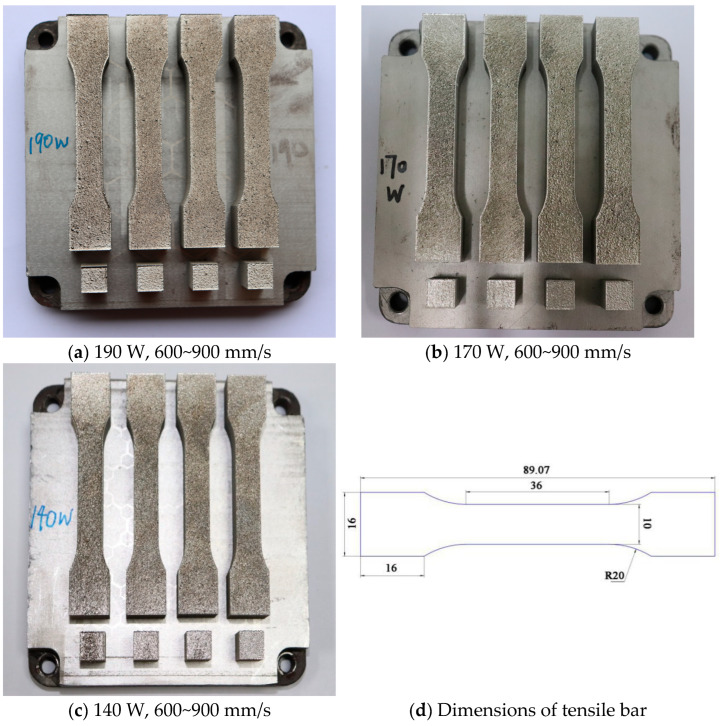
Tensile and metallographic specimens of LPBF-fabricated 316L stainless steel produced at different laser powers (140 W, 170 W, and 190 W) and scanning speeds (600 mm/s to 900 mm/s): (**a**) specimens at 190 W, (**b**) specimens at 170 W, (**c**) specimens at 140 W, and (**d**) dimensions of the tensile bar.

As shown in Figure 1, in this experiment, each substrate was printed with a total length of 89.07 mm tensile parts and a metallographic specimen of 10 mm × 10 mm × 3 mm at laser powers of 190 W, 170 W, and 140 W and scanning speeds of 600 mm/s, 700 mm/s, 800 mm/s, and 900 mm/s. The metal tensile part specimens were designed and formed according to the GB/T 228.1-2021 standard [41]. The formed metal specimens were then taken for wire cutting. Three tensile specimens with a thickness of 3 mm and one metallographic specimen were cut from the specimens with each process parameter. Metallurgical defects are more easily introduced into the bonding area between the upper and lower molten pools, so the tensile properties of the specimens manufactured in the vertical direction are lower than those of the specimens manufactured in the horizontal direction. Therefore, the specimens were manufactured horizontally to obtain higher tensile properties.

### 2.2. Powders

This study used 316L stainless steel powder (Material Technology Innovations Co., Ltd., Guangzhou, China), and its chemical composition is shown in Table 2.

**Figure 2:**
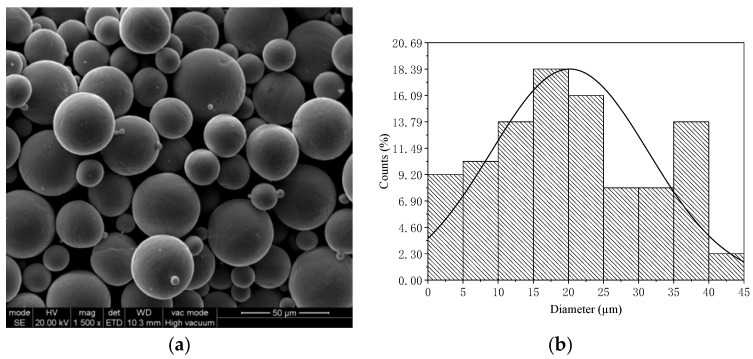
SEM image of morphology (**a**) and diameter statistics (**b**) of 316L stainless steel powder.

The morphology and particle size characteristics of the 316L stainless steel (SS) powder used in this study are shown in Figure 2. The scanning electron microscopy (SEM) image in Figure 2a reveals that the powder particles are predominantly spherical with smooth surfaces, typical of gas-atomized powders. This spherical morphology is essential for ensuring good flowability and high packing density, both of which are critical for the stability and quality of the laser powder bed fusion (LPBF) process. A minimal presence of satellite particles suggests effective powder atomization and classification. The corresponding particle size distribution, shown in Figure 2b, spans approximately 0 to 45 µm and follows a unimodal, moderately right-skewed distribution. Most particles fall within the 15–25 µm range, with a peak near 20 µm and an estimated median particle diameter (D50) of 24.5 µm. This relatively narrow and uniform distribution supports consistent layer deposition, enhances process stability, and contributes to the fabrication of dense, mechanically robust LPBF components.

**Figure 3:**
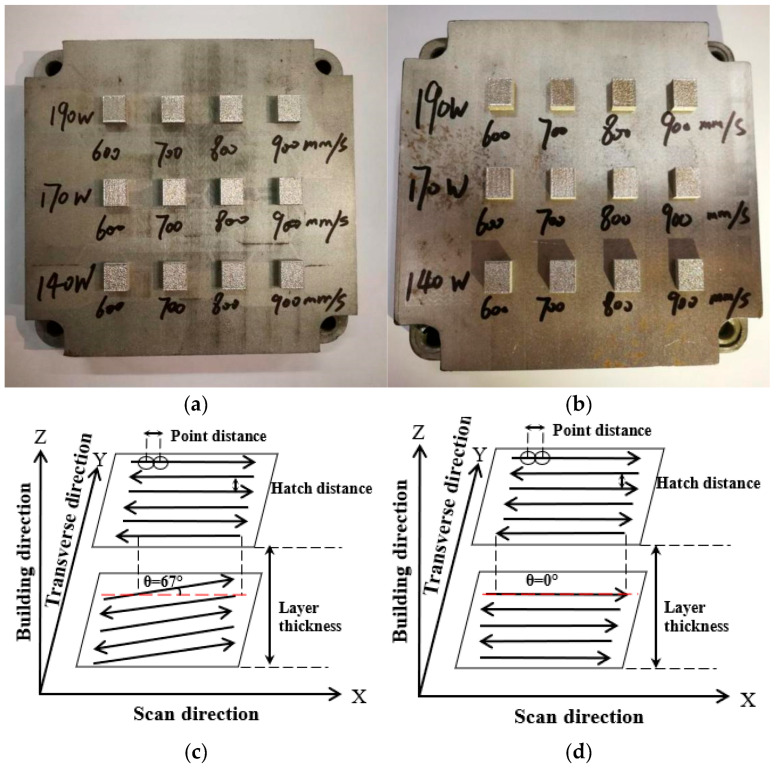
Metallographic specimens and scanning strategies for LPBF-fabricated 316L stainless steel: (**a**) specimen with an interlayer rotation angle of 67°, (**b**) specimen with an interlayer rotation angle of 0°, (**c**) scanning strategy diagram for 67° rotation, and (**d**) scanning strategy diagram for 0° rotation.

In Figure 3a,b, metallographic specimens of 316L stainless steel fabricated through laser printing are presented. Each sample was subsequently cut from the substrate using a wire-cutting machine. The printing parameters included a laser power range of 140–190 W, scanning speeds of 600–900 mm/s, and layer rotation angles of 67° and 0°. Small blocks measuring 8 mm × 10 mm × 3 mm were printed and prepared for microstructural observation. Figure 3c,d illustrate the scanning strategies with layer rotation angles of 67° and 0°, respectively, showcasing the differences in the scanning patterns employed during the LPBF process.

### 2.3. Microstructure Characterization

Block samples measuring 8 mm × 10 mm × 3 mm were printed using different combinations of laser power and scanning speed to observe their microstructural morphology. A stripe partition scanning strategy was employed. The samples were divided into two groups based on the layer rotation angle for analysis. The first group, with a layer rotation angle of 67°, was studied to examine the influence of microstructure on tensile properties. The second group, with a layer rotation angle of 0°, was investigated to analyze the relationship between scanning direction and the shape of the melt pool. This approach provided insights into the effects of scanning strategies on the LPBF process and the resulting material properties.

Porosity was quantitatively measured by metallographic image analysis. For each sample, cross-sectional images were obtained using optical microscopy after standard grinding and polishing. The images were analyzed with ImageJ version 1.47q software, using threshold segmentation to identify and measure pore areas. The areal porosity was calculated as the total pore area divided by the total analyzed area. At least three representative fields of view were evaluated for each processing condition, and the mean porosity was reported. The relative density of the LPBF-fabricated 316L stainless steel specimens was measured using the Archimedes density method.

The phase identification of the fabricated samples was performed using an Ultima IV X-ray powder diffractometer (Rigaku Ltd., Tokyo, Japan) operated at 40 kV and 40 mA with CuKα radiation. Continuous scanning was conducted within a scattering angle range of 30–110° at a rate of 4°/min. The microstructure and texture were examined by Mira3 LC SEM (TESCAN Ltd., Warrendale, PA, USA) equipped with an electron back scatter diffraction (EBSD) detector (Oxford Instruments Ltd., Oxford, UK) at 20 kV and 1.6 nA with 2 μm steps. The texture and mislocation analyses were conducted by the AZcrystal 3.0 software (Oxford Instruments Ltd., Oxford, UK).

Samples for metallographic analysis were prepared following standard procedures, including cutting, grinding, and polishing. Samples were prepared to ensure a flat, smooth surface free of oxide scale, grease, or other contaminants. The specimens were sequentially ground with SiC papers ranging from 600 to 2000 mesh, followed by polishing with W2.5 and W1.5 diamond pastes. Subsequently, they were ultrasonically cleaned in alcohol, rapidly dried, and wrapped in clean cotton prior to hardness testing. The polished samples were etched for 10 min using a solution composed of nitric acid (6 mL), hydrochloric acid (2 mL), and water (10 mL), with the beaker shaken every 3 min to ensure uniform etching. The microstructure of the samples was characterized using a Leica DM3000 optical microscope (Leica Microsystems GmbH, Wetzlar, Germany). Additionally, a Phenom XL scanning electron microscope (SEM) equipped with an energy dispersive spectrometer (EDS) microprobe system was utilized to estimate the elemental distribution and composition of the samples. This comprehensive analysis provided detailed insights into the microstructural and compositional characteristics of the 316L stainless steel specimens.

### 2.4. Mechanical Properties Test

The tensile test was carried out at room temperature using an MTS 831.10 testing machine (MTS Systems Ltd., Eden Prairie, MN, USA) at a speed of 2 mm/min. The mechanical properties of the LPBF-formed parts were tested in accordance with the GB/T 228.1-2010 standard. The strain of the specimen was measured using a VIC-3D™ system (Correlated Solutions, Inc., Columbia, SC, USA). As shown in Figure S3, it is the transverse and longitudinal strain cloud diagram at each stage of stretching by the digital image correlation method.

The Vickers hardness test of metallic materials was conducted in accordance with the GB/T 4340.2-2012 standard. Measurements were performed using an HXD-1000TMSC/LCD Vickers hardness tester (Remet Ltd., Bologna, Italy) under a test load of 200 g and a dwell time of 15 s. For each metallographic specimen, three indentations were made on the cross-section corresponding to the tensile direction, and the average value was reported as the Vickers hardness.

## 3. Results

### 3.1. Processing Parameter and Density

**Figure 4:**
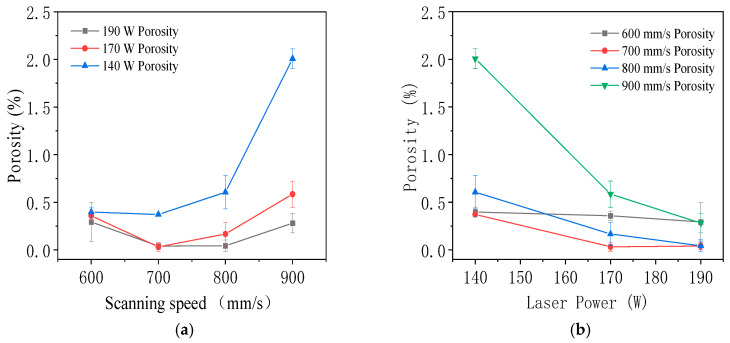
Effect of (**a**) scanning speed and (**b**) laser power on porosity.

As shown in Figure 4a, at constant laser power, porosity initially decreased and then sharply increased with increasing scanning speed. The high porosity observed at 600 mm/s was attributed to excessive energy density, which produced deep melt pools and promoted the formation of keyholes and gas pores. At 700 mm/s and 800 mm/s, porosity was minimized due to a more moderate melt pool depth, which allowed trapped gas to escape. In contrast, scanning at 900 mm/s resulted in the highest porosity, caused by insufficient energy input. This led to incomplete fusion between adjacent melt pools and the formation of cracks at pool boundaries, which evolved into larger pores. Figure 4b shows that porosity generally decreased with increasing laser power. At a scanning speed of 600 mm/s, laser power had minimal effect on porosity. However, at higher scanning speeds, porosity was consistently highest at 140 W and lowest at 190 W, indicating that increased laser power improved melt pool stability and reduced defect formation.

**Figure 5:**
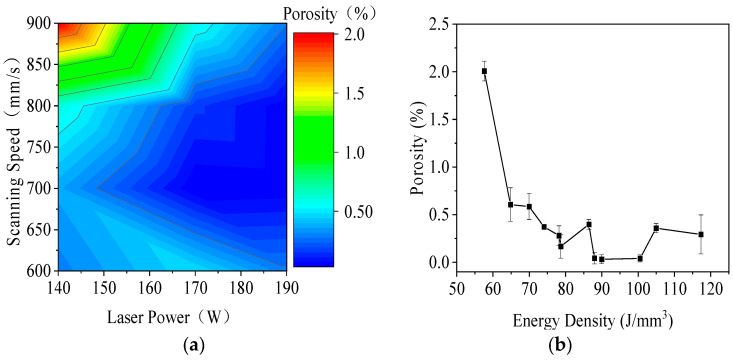
Influence of processing parameters on porosity in LPBF-fabricated 316L stainless steel: (**a**) contour plot of porosity as a function of laser power (140 to 190 W) and scanning speed (600 to 900 mm/s), and (**b**) the relationship between energy density (50 to 120 J/mm^3^) and porosity, showing minimum porosity at 90 to 100 J/mm^3^.

Figure 5 illustrates the influence of processing parameters on porosity in the LPBF process for 316L stainless steel. In Figure 5a, a contour plot shows porosity as a function of laser power (140 to 190 W) and scanning speed (600 to 900 mm/s). Porosity decreased with increasing laser power and decreasing scanning speed. The minimum porosity of approximately 0.04% occurred at 190 W and 700 to 800 mm/s, while the maximum porosity of about 2% appeared at 140 W and 900 mm/s due to insufficient energy input and weak melt pool bonding.

Figure 5b shows the relationship between energy density (50 to 120 J/mm^3^) and porosity. As energy density increased, porosity generally decreased, reaching the lowest values between 90 and 100 J/mm^3^. At both low and high energy densities, porosity exhibited large standard deviations, which were attributed to the random formation of pores and interlayer voids. These results emphasized the importance of optimizing laser power, scanning speed, and energy density to minimize porosity and improve part quality during LPBF processing.

This chapter demonstrated that LPBF-processed 316L stainless steel could achieve high relative densities (up to 99.97%) and very low porosity (<0.05%) under optimized parameters. These characteristics are well-recognized as prerequisites for load-bearing implants, as they minimize the risk of crack initiation and corrosion-related degradation in vivo. Furthermore, Barrionuevo et al. [42] support that high densification in LPBF-fabricated 316L can contribute to a reduction in stress shielding and promote better osseointegration, both of which are highly desirable in orthopedic and dental implants.

### 3.2. Phase Analysis

**Figure 6:**
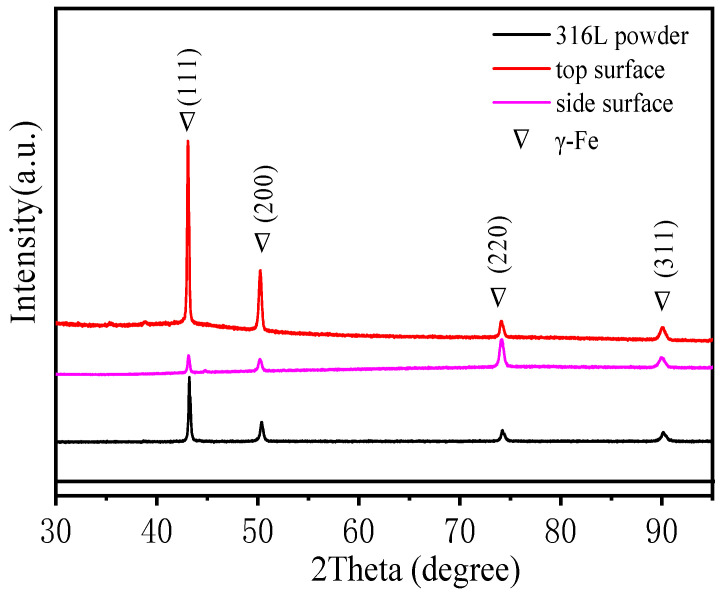
XRD pattern of 316L stainless steel powder and the surfaces of LPBF fabricated samples with 67° layer rotation angle.

Figure 6 presents the X-ray diffraction (XRD) patterns of 316L stainless steel powder and the top and side surfaces of samples fabricated using LPBF. The diffraction peaks corresponding to γ-Fe planes (111), (200), (220), and (311) are labeled. The powder exhibited characteristic peaks consistent with the γ-Fe austenitic phase. The top surface of the LPBF-fabricated sample showed a preferential orientation along the (111) plane, indicated by the increased intensity of that peak, while the side surface exhibited a stronger (220) orientation. These differences reflected anisotropic microstructural development, likely influenced by directional heat dissipation and rapid cooling during fabrication.

Saeidi et al. [39] also observed preferential orientation along the (111) plane in LPBF samples and attributed it to high undercooling and directional solidification that promoted uniform grain growth. AlMangour et al. [43] identified austenite as the dominant phase, with trace ferrite (α-Fe) resulting from high cooling rates. Yakout et al. [44] reported similar austenitic structures with minor martensitic peaks, suggesting the formation of needle-like martensite with a body-centered tetragonal (BCT) structure. In contrast, Wang et al. [45] detected only a single austenitic phase with no evidence of martensitic transformation.

**Figure 7:**
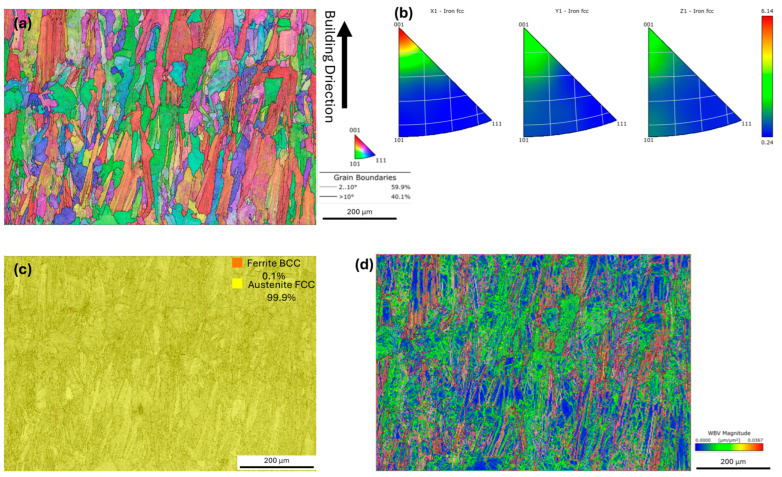
Electron backscatter diffraction (EBSD) analysis of LPBF-fabricated 316L stainless steel: (**a**) inverse pole figure (IPF) map showing grain orientation along the building direction; (**b**) pole figures for the FCC (austenite) phase; (**c**) phase map indicating 99.9% austenite (FCC) and 0.1% ferrite (BCC); (**d**) Weighted Burgers Vector (WBV) misorientation density map, illustrating the distribution of local dislocation density within the microstructure.

Electron backscatter diffraction (EBSD) analysis provided detailed insight into the microstructure of 316L stainless steel fabricated by laser powder bed fusion (LPBF), as shown in Figure 7. The grain size analysis, based on an equivalent circle diameter approach (threshold angle 10°), revealed a mean grain size of 23.4 μm. The inverse pole figure (IPF) map in Figure 7a reveals a strong crystallographic texture along the building direction, with elongated grains that are typical for additively manufactured austenitic stainless steels. The grain boundary analysis shown in Figure 7a indicates that low-angle grain boundaries (LAGBs, with misorientation angles between 2 and 10 degrees) accounted for 59.9% of the total grain boundary length, while high-angle grain boundaries (HAGBs, above 10 degrees) constituted the remaining 40.1%. The dominance of LAGBs reflected the prevalence of subgrain and cellular structures generated by the rapid solidification conditions during LPBF processing. These LAGBs are effective in impeding dislocation motion and promoting work hardening, thereby contributing to the material’s strength and ductility. The pole figures shown in Figure 7b reveal a strong crystallographic texture along the building direction, with the majority of austenite grains preferentially oriented along the <001> direction. This texture developed as a result of the steep thermal gradients and unidirectional heat flow during the laser powder bed fusion process, which promoted epitaxial growth of columnar grains parallel to the building direction. Such a strong <001> texture is known to induce mechanical anisotropy, typically resulting in improved ductility and work-hardening capacity along the build direction, as the <001> axis in FCC austenite facilitates slip and coordinated plastic deformation. The phase map Figure 7c demonstrates that the microstructure consisted of 99.9% austenite (FCC) and only 0.1% ferrite (BCC), confirming the near-complete stabilization of the austenite phase by the combined effects of high cooling rates and nickel enrichment. The weighted Burgers vector (WBV) misorientation density map in Figure 7d illustrates the spatial distribution of local lattice misorientation, which is closely related to dislocation density. Regions with high WBV values indicated higher stored strain and dislocation content, often resulting from the rapid cooling and repeated thermal cycling during layer-by-layer fabrication. A higher density of dislocations, as reflected by elevated WBV values, can significantly enhance the work hardening rate of the material, contributing to increased tensile strength and ductility. The dense network of dislocations impedes the movement of new dislocations during plastic deformation, resulting in more uniform strain distribution and delayed necking under tensile loading.

### 3.3. Metallography of 67° Interlayer Rotation

**Figure 8:**
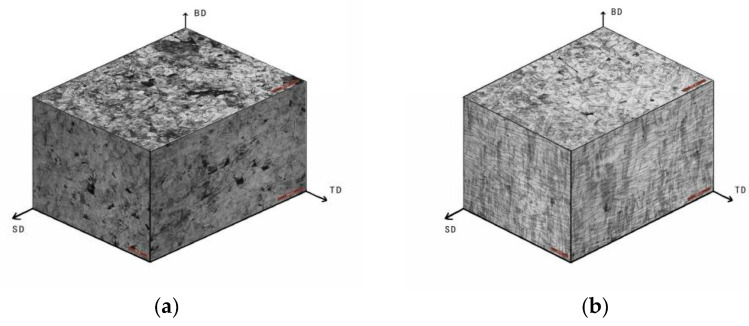

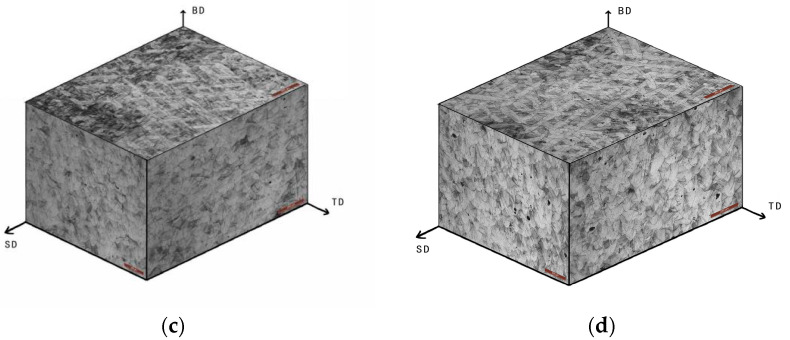
Stereo metallographic images of 316L stainless steel specimens with laser power of 140 W and scanning speed of (**a**) 900 mm/s, (**b**) 800 mm/s, (**d**) 700 mm/s, and (**d**) 600 mm/s with 67° interlayer rotation.

The interlayer rotation angle of 67° was applied in this test to generate anisotropic molten pools and reduce residual stress caused by rapid cooling during LPBF processing. As shown in Figure 8, the three-dimensional metallographic images captured under a laser power of 140 W and scanning speeds ranging from 600 mm/s to 900 mm/s illustrate the relationship among the building direction (BD), scanning direction (SD), and transverse direction (TD). At 140 W and 900 mm/s, a high number of unmelted powder particles resulted in numerous pores and distorted scanning tracks. In contrast, at 140 W and 600 mm/s, the molten pool contained only a few circular pores near the bottom, and the scanning tracks appeared clear and straight. The intersection angle between successive layers was visibly aligned with the 67° rotation setting.

**Figure 9:**
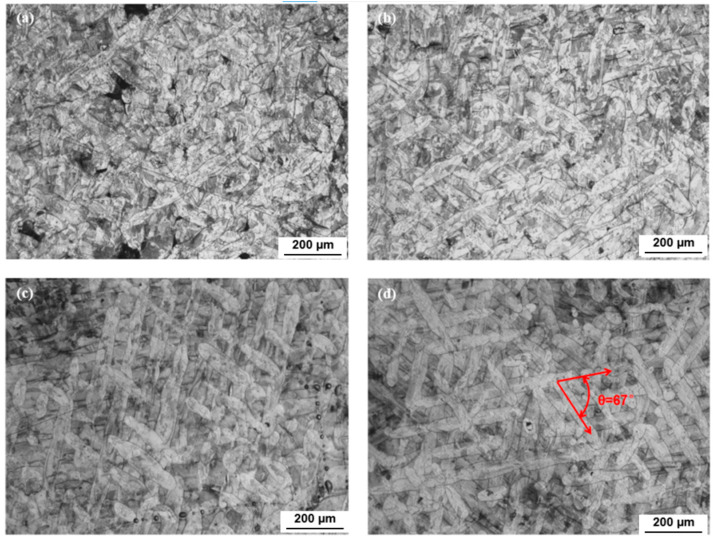
Top surface metallographic images of 316L stainless steel specimens fabricated by LPBF with an interlayer rotation angle of 67° at a laser power of 140 W and scanning speeds of (**a**) 900 mm/s, (**b**) 800 mm/s, (**c**) 700 mm/s, and (**d**) 600 mm/s, showing variations in melt track width, porosity, and scanning track morphology.

As shown in Figure 9, the top surfaces of specimens with an interlayer rotation angle of 67° exhibited melt path widths of approximately 45 μm, 50 μm, 57 μm, and 61 μm in Figure 9a through Figure 9d, respectively. The melt path width increased as the scanning speed decreased, reflecting higher energy input. The angle between overlapping and intersecting tracks was clearly about 67°, consistent with the applied layer rotation. In Figure 9a, the specimen showed high porosity and irregular, tortuous melt tracks. This was caused by low energy density, which led to incomplete melting of the powder and the formation of defects between layers. The insufficient energy also prevented the laser from penetrating the melt stratification zone, resulting in weak interlayer bonding. Furthermore, due to surface tension at the gas–liquid interface, the molten pool rapidly spheroidized during solidification. In contrast, Figure 9d showed a specimen produced with higher energy density, where the melt tracks were straight, uniform in width, and free of defects such as spheroidization and unmelted powder.

As shown in Figure S7, when the interlayer rotation angle was set to 67°, the scanning tracks and cross-sections intersected at varying angles, resulting in molten pools with diverse shapes and sizes. At low scanning speeds, the molten pools were generally deeper and wider, with fewer pores. In contrast, high scanning speeds produced shallower molten pools with increased porosity. These metallographic images were intended for use as datasets in neural network training and validation. The side views of the specimens revealed irregular variation in the depth and width of molten pools. This was due to the 67° rotation applied between successive layers. When the scan track of a given layer was perpendicular to the cross-section, the corresponding molten pool appeared largest and exhibited a “fish scale” morphology. Conversely, when the track was parallel to the cross-section, the molten pool appeared narrower and more strip-like. As a result, the depth and thickness of the molten pools in the 67° group lacked consistent patterns when viewed from the side. Therefore, specimens with a 0° interlayer rotation were also studied to more directly evaluate the influence of laser power and scanning speed on molten pool geometry.

### 3.4. Metallography of 0° Interlayer Rotation

**Figure 10:**
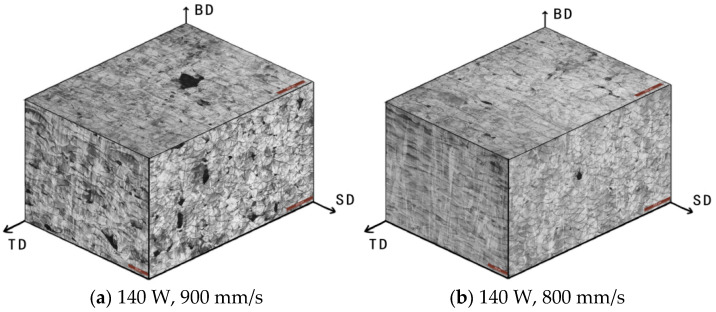

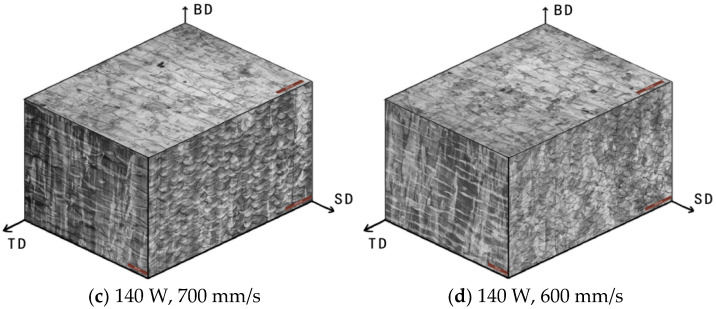
Three-dimensional metallographic images of 316L stainless steel specimens fabricated with an interlayer rotation angle of 0° at a laser power of 140 W and scanning speeds of (**a**) 900 mm/s, (**b**) 800 mm/s, (**c**) 700 mm/s, and (**d**) 600 mm/s, showing melt pool morphology on different planes.

As shown in Figure 10, the specimens were fabricated under conditions of 0° interlayer rotation, 140 W laser power, and scanning speeds ranging from 600 mm/s to 900 mm/s. The transverse direction (TD), scanning direction (SD), and building direction (BD) defined the spatial orientation of the sample. The BD-SD plane, which was parallel to the scanning track, was referred to as the parallel plane; the BD-TD plane, perpendicular to the scanning direction, was defined as the vertical plane; and the TD-SD plane represented the top plane. When scanning was performed in a single direction, the molten pool appeared strip-shaped on the parallel plane and fish scale-shaped on the vertical plane. Under the condition of 140 W and 900 mm/s, the porosity was highest. This was attributed to the presence of unmelted particles and spheroidization, which obstructed flow and disrupted the formation of continuous scanning tracks, resulting in distorted and discontinuous melt paths.

**Figure 11:**
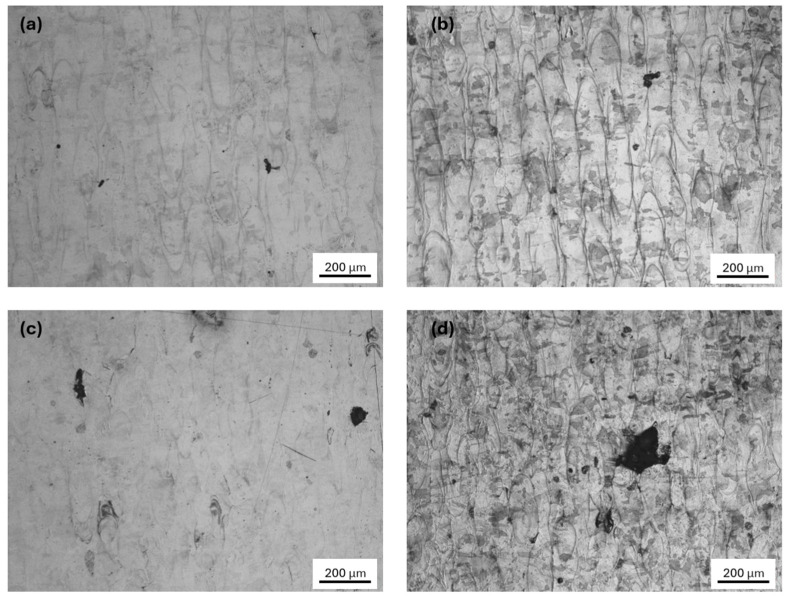
Top surface (SD-TD plane) metallographic images of 316L stainless steel specimens fabricated with an interlayer rotation angle of 0° at a laser power of 140 W and scanning speeds of (**a**) 600 mm/s, (**b**) 700 mm/s, (**c**) 800 mm/s, and (**d**) 900 mm/s, showing variations in scanning track width and porosity.

As shown in Figure 11, the images depict the top surface (SD-TD plane) of metallographic specimens fabricated with an interlayer rotation angle of 0°. The average scanning track widths in Figure 11a through Figure 11d were approximately 88 μm, 79 μm, 77 μm, and 68 μm, respectively. The results indicated that, under constant laser power, the scanning track width decreased as scanning speed increased. In Figure 11d, the specimen shows a large lack of fusion (LoF) pores, suggesting insufficient energy input at higher scanning speeds. In general, the top surface track widths under 0° interlayer rotation were larger than those observed at 67°. This was because the tracks produced by unidirectional scanning at 0° remained in the same plane and were well aligned, leaving no visible gaps. In contrast, under 67° rotation, the scanning tracks from successive layers did not lie in the same plane due to the angular offset. As a result, when the surface was ground in a single direction for imaging, only a partial cross-section of the top of each melt pool was revealed, making the measured track widths appear smaller than their actual dimensions due to partial coverage from overlapping layers.

Figures S8–S10 are metallographic images of the vertical surface, and the molten pool is “fish scale-like”. As shown in Figure S6, the metallographic image of the parallel surface, the molten pool is strip-like. Five images are counted for each working condition, five molten pools are counted in each image, and the average value is taken.

**Figure 12:**
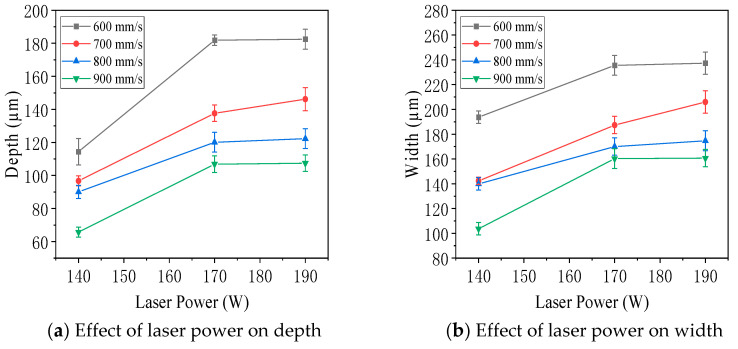

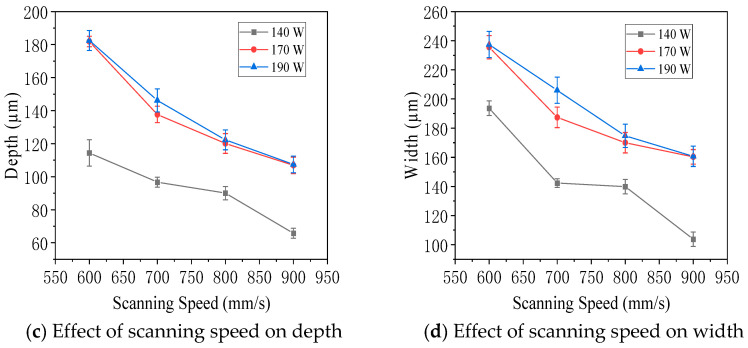
Effect of laser power and scanning speed on the depth and width of molten pools on the vertical surface of 316L stainless steel specimens with an interlayer rotation angle of 0°: (**a**,**b**) show variations with laser power; (**c**,**d**) show variations with scanning speed.

Figure 12 presents the measured depth and width of molten pools on the vertical plane of specimens fabricated with an interlayer rotation angle of 0° under varying laser power and scanning speed. As shown in Figure 12a,b, both the depth and width of the molten pools increased with increasing laser power and gradually stabilized. Figure 12c,d showed that, with constant laser power, increasing the scanning speed led to a decrease in both the depth and width of the molten pools. These trends indicated that higher energy input, achieved through increased laser power or reduced scanning speed, promoted deeper and wider melt pools, enhancing material fusion and process stability.

**Figure 13:**
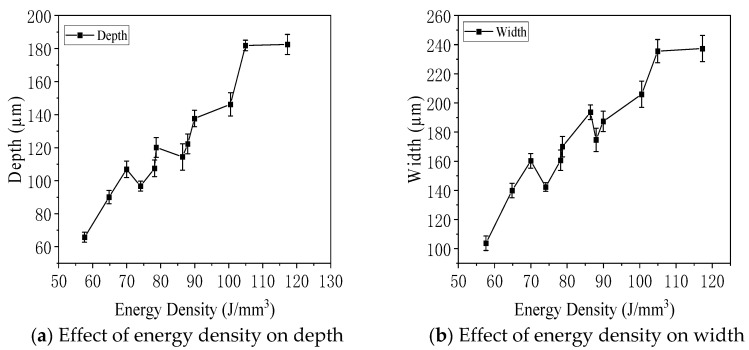

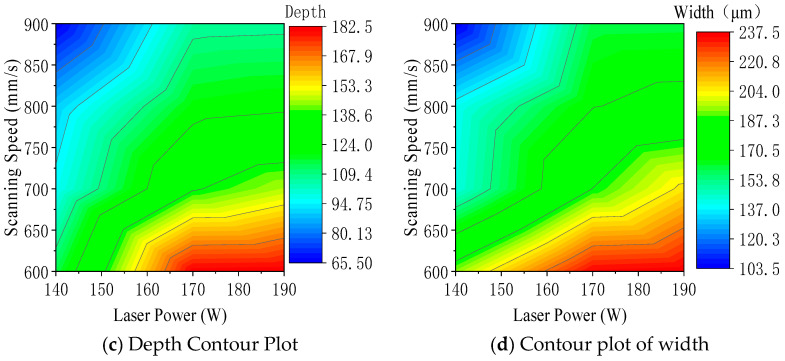
Effect of energy density on molten pool geometry in the vertical plane of 316L stainless steel specimens with an interlayer rotation angle of 0°: (**a**) depth versus energy density, (**b**) width versus energy density, (**c**) depth contour plot, and (**d**) width contour plot.

Figure 13 illustrates the influence of energy density on the depth and width of molten pools observed on the vertical plane of specimens fabricated with an interlayer rotation angle of 0°. As shown in Figure 13a,b, both the depth and width of the molten pools increased with increasing energy density and then gradually stabilized. Contour plots in Figure 13c,d further confirmed this trend. Under the condition of 190 W laser power and 600 mm/s scanning speed, corresponding to an energy density of 117.28 J/mm^3^, the molten pool exhibited the largest average width of 237 μm and depth of 183 μm. In contrast, at 140 W and 900 mm/s, with an energy density of 57.61 J/mm^3^, the smallest average width and depth were observed—104 μm and 66 μm, respectively. These results demonstrated that higher energy density promoted deeper and wider molten pools, improving material melting and bonding.

**Figure 14:**
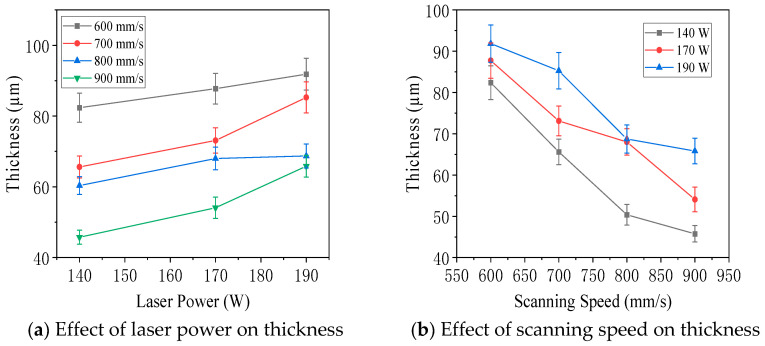

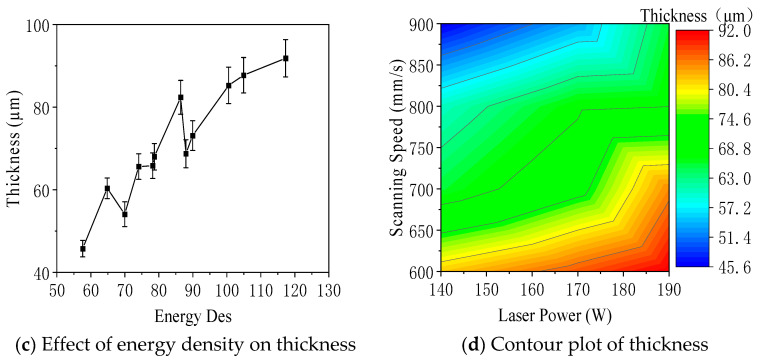
Effect of laser power, scanning speed, and energy density on the molten pool thickness of 316L stainless steel specimens in the parallel plane with an interlayer rotation angle of 0°: (**a**) thickness versus laser power, (**b**) thickness versus scanning speed, (**c**) thickness versus energy density, and (**d**) thickness contour plot. Microstructures.

Figure 14 illustrates the effect of laser power, scanning speed, and energy density on the thickness of molten pools observed on the parallel surface of specimens with an interlayer rotation angle of 0°. As shown in Figure 14a, when the scanning speed was held constant, the molten pool thickness increased with higher laser power. Figure 14b showed that, under constant laser power, the thickness decreased as the scanning speed increased. Figure 14c demonstrated a positive correlation between energy density and molten pool thickness. The contour plot in Figure 14d summarizes these trends. The maximum thickness was 92 µm at 190 W and 600 mm/s, corresponding to an energy density of 117.28 J/mm^3^, while the minimum thickness was 46 µm at 140 W and 900 mm/s, with an energy density of 57.61 J/mm^3^. The thickness of the molten pool on the parallel surface was generally smaller than the depth observed on the vertical surface. This was because the parallel surface did not intersect the central axis of the molten pool in the vertical plane, resulting in lower measured values. Therefore, the parallel surface thickness was not used as the standard metric for molten pool geometry.

### 3.5. Defects

**Figure 15:**
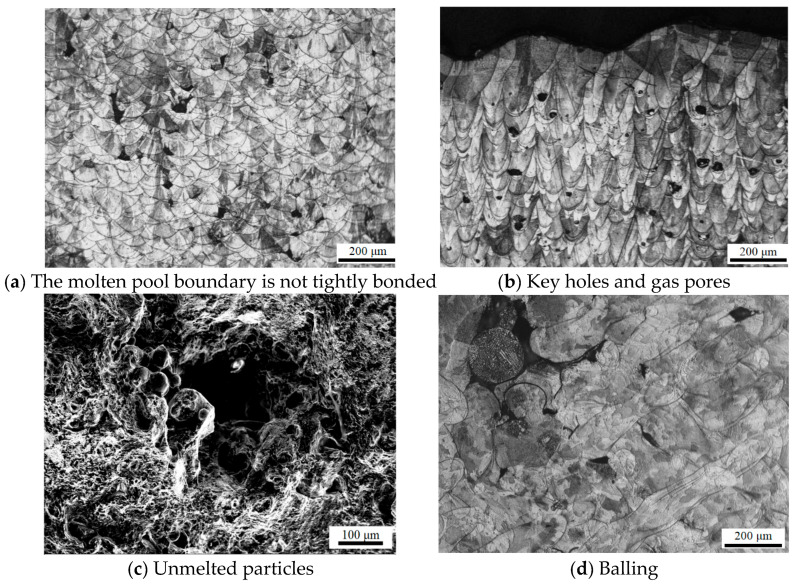
Common defect types observed in LPBF-fabricated 316L stainless steel: (**a**) lack of fusion due to low energy density and poor bonding at molten pool boundaries, (**b**) keyhole and gas pore formation caused by excessive energy input and gas entrapment, (**c**) unmelted powder particles acting as crack initiation sites, and (**d**) balling at the top of the molten pool due to surface tension effects and spatter-induced instability.

As shown in Figure 15, four types of defects were observed in the molten pool region. Figure 15a shows a defect caused by excessive scanning speed and low energy density. Under these conditions, the molten pool boundary was not properly bonded, and residual stress led to crack initiation at the boundary, eventually forming large-area voids. These regions were often accompanied by partially unmelted powder particles, further weakening the material. Figure 15b depicts pore defects formed under low scanning speed and high energy density. In this case, the molten pool was deep, and nitrogen from the protective atmosphere dissolved into the melt due to intense convection. As the laser moved on and rapid solidification occurred, the gas was trapped before it could escape, forming spherical pores near the bottom of the molten pool. These defects were typically small and remained confined within the melt zone. Figure 15c revealed that unmelted powder particles embedded within the solidified material acted as crack initiation points, leading to the formation of larger pores. These unmelted particles disrupted the metallurgical continuity and significantly reduced mechanical integrity. Figure 15d showed a spheroidization phenomenon at the top of the uppermost molten pool layer. This defect originated from two primary causes: poor wettability between the molten metal and the solid substrate, which depended on surface tension, and droplet splashing during the melting process. The spheroidized particles failed to coalesce and left behind internal voids. These spherical defects degraded mechanical properties, increased surface roughness, and posed risks to the powder spreading mechanism. Specifically, they elevated friction against the recoater blade, potentially causing mechanical wear or damage to the equipment.

Defects in additively manufactured 316L stainless steel, such as pores, unmelted powder particles, lack-of-fusion regions, and balling, have important implications not only for mechanical integrity but also for the biological performance of implantable devices. High porosity and surface-connected voids may serve as initiation sites for crack propagation under cyclic physiological loads, potentially compromising implant longevity and increasing the risk of failure in vivo [46]. Moreover, interconnected pores and rough defect surfaces can accelerate corrosion processes in the physiological environment, which may lead to the release of metallic ions that can trigger adverse tissue reactions or inflammatory responses [19].

From a biomaterials standpoint, the presence and morphology of defects can significantly affect the tissue response at the implant–tissue interface. For example, surface-connected pores may alter protein adsorption and cellular adhesion, which are critical for successful osseointegration and long-term implant stability [47]. While controlled surface porosity can sometimes promote bone ingrowth and mechanical interlocking, excessive or irregular defects may instead lead to fibrous encapsulation or local inflammation, undermining the desired biointegration [48].

Thus, minimizing the size, number, and connectivity of process-induced defects is crucial for optimizing both the mechanical and biological outcomes of LPBF-fabricated 316L implants. A comprehensive understanding of the relationships among processing parameters, defect formation, and subsequent biological interactions is necessary to ensure safe and reliable performance of metallic biomaterials in clinical applications [49].

### 3.6. Grain Morphology

**Figure 16:**
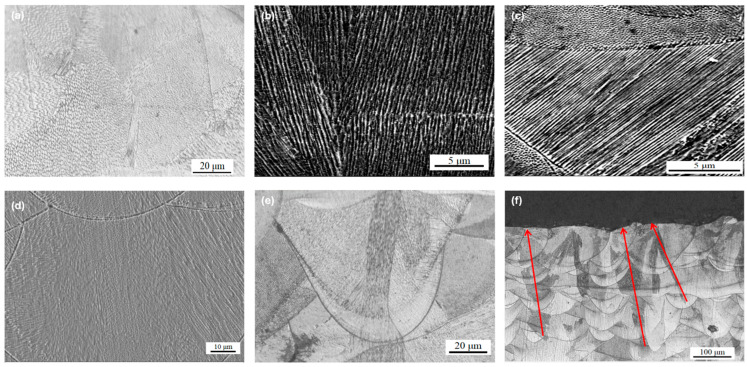
Microstructural morphology and growth behavior of 316L stainless steel fabricated by laser powder bed fusion: (**a**) cellular crystal cross-section, (**b**) columnar crystal morphology, (**c**) columnar grains growing perpendicular to the molten pool boundary, (**d**) columnar grains growing at an angle to the boundary, (**e**) epitaxial growth of columnar grains across multiple layers, and (**f**) etched structure showing the direction of heat conduction and grain growth. Red arrows indicate the direction of columnar grain growth.

In Figure 16a, the honeycomb cellular structure represents the cross-sectional view of columnar crystals, while Figure 16b displays the longitudinal morphology of the columnar crystals. Figure 16c,d show that the columnar crystals grew either perpendicular to or at an angle to the molten pool boundary. This growth followed a non-uniform nucleation and crystallization mechanism, driven by thermal gradients. Heat within the molten pool primarily diffused from the melt zone toward the previously solidified material and the substrate, promoting directional solidification along the path of the steepest temperature gradient.

Figure 16e,f illustrate that the columnar crystals extended across multiple molten pool layers. During the LPBF process, melting of the upper layer caused heat to conduct downward into the solidified austenite below, triggering recrystallization along the thermal path. Due to the high thermal conductivity and substantial supercooling, the resulting recrystallized grains were fine. The direction opposite to heat conduction marked the epitaxial growth path of the columnar grains, which became visible after etching. This observation was consistent with the findings of Qiu et al. [50], who reported that epitaxial grain growth could span more than ten layers. Ma et al. [51] similarly visualized epitaxial columnar grains using three-dimensional imaging and discussed the influence of processing conditions on their length and width. Zhong et al. [52] concluded, based on high-magnification electron microscopy, that the grain morphology consisted of vertically oriented columnar crystals and horizontally oriented cellular crystals in the cross-section.

**Figure 17:**
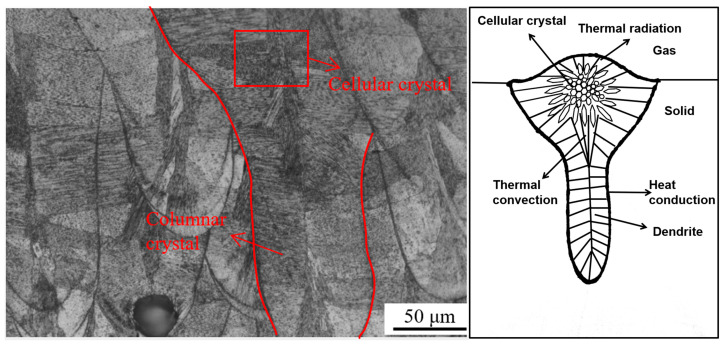
Typical morphology of a molten pool in LPBF-fabricated 316L stainless steel: the left image shows the overall shape of the molten pool with columnar crystals growing perpendicular or at an angle to the boundary due to thermal gradients; the right image highlights cellular crystals in the center and the effect of heat conduction and convection on grain growth orientation and solidification behavior.

Figure 17 shows a typical morphology of the molten pool formed during the LPBF process. Heat conduction was the primary mode of heat dissipation, resulting in a high cooling rate at the molten pool boundary. Consequently, the growth direction of the columnar crystals near the boundary was either perpendicular to the boundary or aligned along the steepest temperature gradient. Within the molten pool, heat convection occurred, and due to the influence of gravity, the convective flow was predominantly vertical. This internal convection altered the growth direction of columnar crystals, causing variation across the melt pool region.

Cellular crystals were observed in the central region of the molten pool. The gradual increase in the length of their axes from the center outward indicated that heat dissipated from the core toward the surrounding material. Liu et al. [53] reported that columnar crystal morphology varied with height due to repeated thermal cycling. Heat conducted downward from the upper layers caused the lower layers to experience grain coarsening and lamellar structure formation, with the grain axis oriented along the build direction. Brytan [54] also observed a honeycomb columnar structure, where the overlapping of molten pools caused submicron grains to shift their growth direction, exhibiting epitaxial columnar growth across multiple layers.

**Figure 18:**
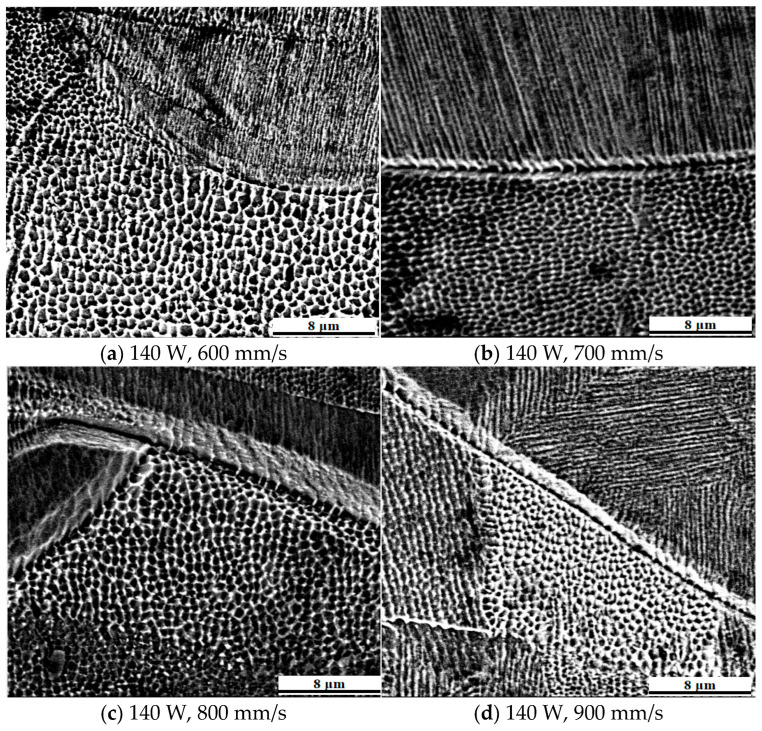
Effect of scanning speed on cellular diameters in LPBF-fabricated 316L stainless steel: (**a**) 600 mm/s, (**b**) 700 mm/s, (**c**) 800 mm/s, and (**d**) 900 mm/s, showing a decrease in average cellular grain diameter from 0.93 µm to 0.55 µm with increasing scanning speed due to higher cooling rates and lower energy input.

Figure 18 shows the cellular austenitic structure of 316L stainless steel specimens fabricated at different scanning speeds. The average diameters of the cellular grains in Figure 18a–d were 0.93 µm, 0.89 µm, 0.66 µm, and 0.55 µm, respectively. As scanning speed increased, the diameters decreased due to a reduction in energy input and an increase in cooling rate. Lower scanning speeds provided more thermal energy and longer solidification times, allowing grains to grow larger. In contrast, rapid solidification at higher scanning speeds led to the formation of finer grains. Both cellular and columnar grains were observed in the specimens, and they belonged to the same austenitic phase (γ-Fe). These two morphologies formed under different thermal conditions during solidification. Columnar grains developed along steep and directional thermal gradients, typically growing epitaxially in the build direction. Cellular grains formed under conditions of moderate thermal gradient and higher undercooling, often within the center of the molten pool. While they appear distinct, cellular and columnar structures can transition within a single grain as local thermal gradients vary. In some cases, the cellular appearance may simply reflect a transverse view of a columnar grain. The grain boundaries were enriched with chromium and molybdenum, elements that resist corrosion and are less susceptible to etching, thus outlining the microstructure. These observations aligned with previous findings that prolonged thermal cycles promoted element diffusion, grain boundary refinement, and transitions between grain morphologies. The current study confirmed that the observed needle-like structures corresponded to differently oriented austenitic grains, as demonstrated by EBSD mapping. The EBSD results showed that over 99.9% of the matrix was composed of austenitic iron, providing direct microstructural evidence in support of the XRD findings, which indicated a predominantly single austenitic phase.

In addition, due to the grain characteristics, the more equiaxed crystals there are in the sample structure, the higher the tensile strength and the lower the elongation. Conversely, the more columnar crystals there are, the higher the elongation and the lower the tensile strength. Shang et al. [55] found that as the scanning speed decreases, the average diameter of the cellular crystals and the distance between the columnar crystals will increase.

According to the Johnson–Mehl formula [56]:

(2) $$P\left(t\right)=k{\left(\frac{N}{v}\right)}^{\frac{3}{4}}$$

In the formula, k represents a constant, ∆T represents the material undercooling which is proportional to the cooling rate, P(t) represents the number of nuclei, N represents the nucleation rate which is proportional to exp(−1/(∆T^2^)), and v represents the grain growth rate which is proportional to ∆T. Both N and v are proportional to ∆T. The growth rate of the former is much greater than that of the latter. In the case of a large undercooling of the laser-melted powder, many nuclei are formed in the molten pool. On the other hand, the rapid cooling process inhibits the growth of the grains, resulting in fine grains.

**Figure 19:**
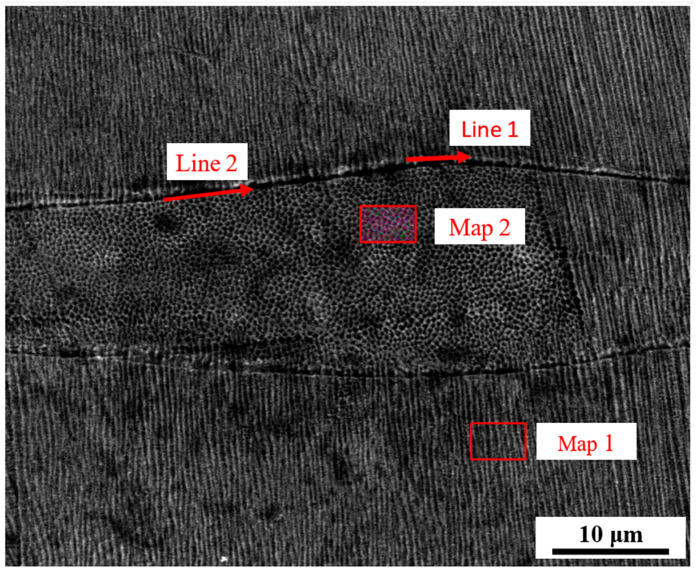
Elemental analysis area and microstructure of 316L SS LPBF samples.

Figure 19 and Table 3 present the elemental distribution and microstructural characteristics of 316L stainless steel specimens fabricated by laser powder bed fusion. Elemental mapping and line scans were performed on both cellular and columnar grain regions. As shown in Map 1 and Map 2 of Figure 19, the chemical compositions of the columnar and cellular crystals were broadly similar. However, the grain boundaries of the cellular regions exhibited pronounced enrichment of chromium and nickel, consistent with solute segregation during rapid solidification. These elements are known to stabilize the austenitic phase and resist corrosion, which also makes the boundaries less responsive to chemical etching. This phenomenon explained the distinct white outlines observed around the cellular grains.

Table 3 provides quantitative results from energy dispersive spectroscopy (EDS). The grain boundaries in Map 1 and Map 2 showed elevated concentrations of chromium (13.73 wt% and 11.96 wt%) and nickel (26.64 wt% and 31.98 wt%), confirming microsegregation. Line 1 and Line 2 data show that the molten pool boundaries were enriched in carbon (5.67 wt% and 3.21 wt%), suggesting the formation of carbides at these locations. Carbides are typically hard and brittle, which can serve as crack initiation sites, especially under residual stress conditions during solidification and cooling. These carbide-rich regions were more susceptible to corrosion and fracture, reducing mechanical performance. This aligned with observations at higher scanning speeds, where many pores were located along molten pool boundaries. The SEM and EDS elemental maps in the larger area in Figure S4 demonstrated a uniform distribution of Fe, Ni, Mo, and Cr throughout the microstructure of the LPBF-fabricated 316L stainless steel.

Microsegregation and repeated thermal cycling in LPBF can enhance diffusion, refine grain boundaries, and contribute to complex microstructural evolution. In this study, the distinction between grain interiors and boundaries, in terms of both chemistry and morphology, emphasized the significant role of solidification dynamics in determining final material properties. Qiu et al. [50] found that there was no obvious difference in the elemental composition between the top and bottom of the molten pool. The columnar crystal structure at the bottom of the molten pool was not due to chemical factors but to different cooling rates.

The grain morphology observed in the LPBF-fabricated 316L stainless steel samples had a significant positive impact for implant applications. The microstructural analysis revealed predominantly fine, cellular, and columnar austenitic grains, which are characteristic of rapid solidification and high thermal gradients in the LPBF process. This refined grain structure was found to enhance both mechanical strength and ductility compared to conventionally processed 316L stainless steel. Such improvements are crucial for load-bearing implants, as they contribute to greater resistance to fatigue and fracture during physiological loading. Additionally, the presence of fine grains increases grain boundary area, which can hinder crack propagation and improve overall toughness—properties essential for the long-term reliability of biomedical implants. Recent studies [22,57] have also suggested that the unique columnar and cellular grain morphology produced by LPBF can promote favorable cellular responses at the tissue–implant interface, further supporting osseointegration and implant stability. Therefore, the grain morphology obtained in this study not only provided mechanical benefits but also reinforced the biological performance required for successful implant integration.

### 3.7. Mechanical Properties

**Figure 20:**
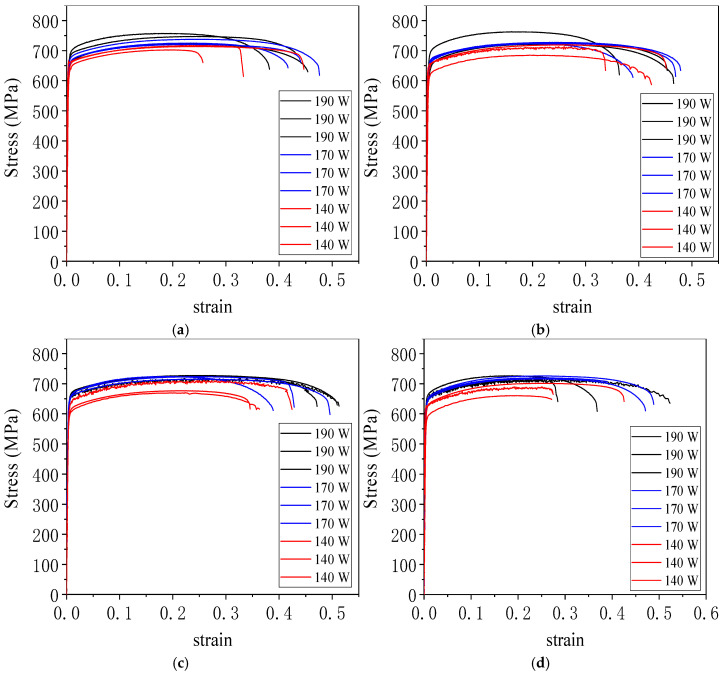
Engineering stress–strain curves of 316L stainless steel specimens fabricated by LPBF under varying process parameters: (**a**) 600 mm/s, (**b**) 700 mm/s, (**c**) 800 mm/s, and (**d**) 900 mm/s scanning speeds at different laser powers, showing the influence of process conditions on tensile behavior.

Figure 20 presents the engineering stress–strain curves of 316L stainless steel specimens fabricated by laser powder bed fusion under varying laser powers and scanning speeds. All specimens showed continuous plastic deformation without a distinct yield point, a behavior characteristic of austenitic stainless steels processed by rapid solidification. The overall trend indicated that higher energy input—achieved by lower scanning speeds or higher laser powers—resulted in greater tensile strength and elongation. Figure S5 shows the tensile parts and metallographic specimens after cutting the 3D printed specimens on the substrate. Figure S6 shows the fractured tensile part.

Figure 20a displays the curves for specimens fabricated at a scanning speed of 600 mm/s. These samples showed the highest tensile strengths and the longest strain ranges before fracture, reflecting dense microstructures and strong interlayer bonding. The elastic region in the stress–strain curves appears as a very short, steep initial segment due to the inherently small elastic strain range of 316L stainless steel compared to its extensive plastic deformation. Figure 20b shows the results for specimens produced at 700 mm/s. Although slightly reduced compared to 600 mm/s, the mechanical response remained strong, with moderate strain hardening and ductility. In Figure 20c, the specimens fabricated at 800 mm/s exhibit a further decrease in tensile strength and elongation. The curves began to steepen, indicating more brittle behavior due to reduced energy input and the onset of microstructural defects. Figure 20d represents specimens printed at 900 mm/s, which showed the lowest mechanical performance across all conditions. These curves ended abruptly, corresponding to early failure likely caused by a lack of fusion, higher porosity, and unmelted powder particles.

The differences among the curves highlighted the strong dependence of mechanical properties on process parameters, with energy input directly influencing grain morphology, defect formation, and fracture resistance. These observations were consistent with fracture surface analyses and porosity trends discussed in related sections of the study.

As shown in Table 4, the tensile strength and Vickers hardness of the 316L specimens formed by laser melting in the selected area under different process parameters in this experiment are shown, and the error is the standard deviation.

Table 4 summarizes the mechanical properties of 316L stainless steel specimens fabricated by laser powder bed fusion under twelve different combinations of laser power and scanning speed. The table includes values for energy density, ultimate tensile strength, and Vickers hardness, with each entry representing the average of three specimens and the accompanying standard deviation.

At 190 W, the specimen fabricated at 600 mm/s achieved a tensile strength of 736.72 MPa and a Vickers hardness of 242.06 HV_0.2_, corresponding to an energy density of 117.28 J/mm^3^. As the scanning speed increased to 700 mm/s, the energy density decreased to 100.53 J/mm^3^, but the hardness improved to 250.10 HV_0.2_, and the tensile strength remained high at 734.10 MPa, indicating a favorable balance of energy input and solidification dynamics. At 800 mm/s and 900 mm/s, the tensile strengths decreased to 724.01 MPa and 722.10 MPa, respectively, while hardness values also declined slightly, reflecting less stable molten pool formation and higher porosity at reduced energy input.

At 170 W, similar trends were observed. The specimen at 600 mm/s showed a tensile strength of 727.70 MPa and a hardness of 248.17 HV_0.2_, with an energy density of 104.94 J/mm^3^. With increasing scanning speed, tensile strength gradually declined from 723.81 MPa at 700 mm/s to 718.80 MPa at 900 mm/s. Hardness also dropped from 243.74 HV_0.2_ to 222.03 HV_0.2_, suggesting insufficient energy delivery at higher scanning speeds.

For 140 W, the lowest energy input conditions were applied. The specimen at 600 mm/s achieved a tensile strength of 707.10 MPa and a hardness of 224.87 HV_0.2_. As scanning speed increased to 700 mm/s, 800 mm/s, and 900 mm/s, tensile strength declined progressively from 699.88 MPa to 688.88 MPa, while hardness fluctuated modestly, with a minimum of 218.73 HV_0.2_ at 700 mm/s. These results indicated that low power combined with high scanning speed resulted in insufficient melting, reduced bonding between layers, and decreased mechanical performance.

Overall, Table 4 demonstrated that both tensile strength and hardness were positively correlated with energy density up to an optimal range. Excessively high or low energy inputs led to degraded performance, either due to overheating and spatter or insufficient fusion. The data highlighted the importance of tuning process parameters to achieve a stable melt pool, low porosity, and refined grain structure, all of which are critical for enhancing the mechanical integrity of LPBF-fabricated components.

**Figure 21:**
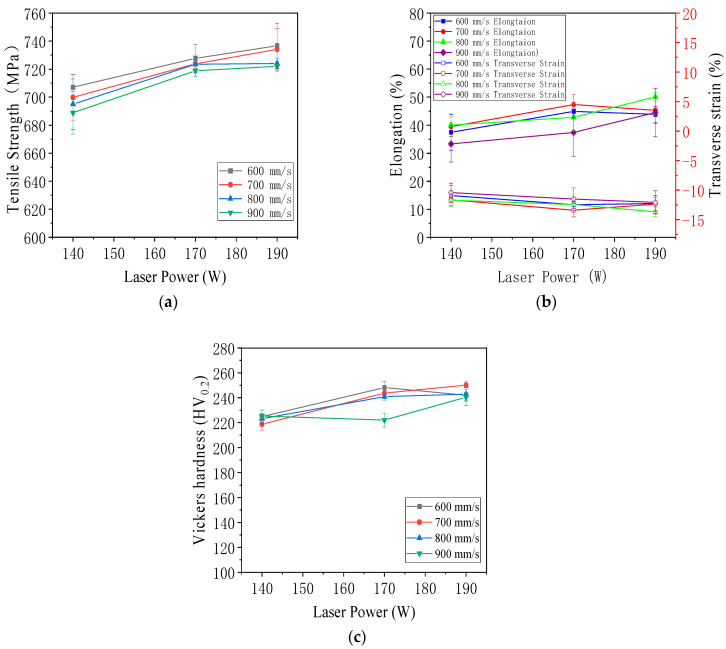
(**a**) Effect of laser power on ultimate tensile strength. (**b**) Effect of laser power on elongation and transverse strain. (**c**) Effect of laser power on Vickers hardness.

Figure 21a shows the effect of laser power on the ultimate tensile strength of LPBF-formed tensile specimens. The median value is the average of three groups of specimens, and the error is the standard deviation. The data and images show that at a certain scanning speed, the tensile strength of LPBF-formed tensile specimens increases with the increase in laser power. The standard deviation of the tensile strength at 140 W is large. The laser power is insufficient, and many holes are formed inside the tensile part. The size, number, and position of these pores are random and uncontrollable, resulting in a large standard deviation at low power. Under the process of laser power of 140 W and scanning speed of 900 mm/s, the lowest tensile strength of this experiment can be obtained, which is 660 MPa, and the energy density of this process is also the lowest in this experiment. Under the process of laser power of 190 W and scanning speed of 700 mm/s, the maximum tensile strength of this experiment can be obtained, which is 763 MPa, and the Vickers hardness obtained under this process parameter is also the largest. The maximum tensile strength obtained in this experiment is much higher than the 596 MPa of cast 316L stainless steel [59] and the 625 MPa of 316L stainless steel [60]. This result provides strong data support for using laser 3D printed 316L stainless steel to manufacture load-bearing parts.

Figure 21b shows the effect of laser power on the elongation and transverse strain of LPBF-formed tensile specimens. The median value is the average of three groups of specimens, and the error is the standard deviation. Under the process of 140 W and 900 mm/s, the minimum elongation of this test is 33.3%, and the minimum transverse strain is −10.4%. Under the process of 190 W and 800 mm/s, the maximum elongation of this test is 50.1%, and the maximum transverse strain is −13.7%. When the scanning speed is constant, the elongation of the tensile specimen formed by LPBF has a slight downward trend with the decrease in laser power, but this trend is not obvious. When the scanning speed is constant, as the laser power decreases, the absolute value of the transverse strain of the tensile specimen formed by LPBF also tends to decrease slightly. Therefore, the elongation and the absolute value of the transverse strain are positively correlated.

As shown in Figure 21c, the effect of laser power on the Vickers hardness of LPBF-formed tensile specimens, the median value is the average of three groups of specimens, and the error is the standard deviation. The scanning speed is 900 mm/s, and the Vickers hardness of 140 W is slightly higher than that of 170 W. This is because the high scanning rate molten pool is shallow, which is prone to interlayer shedding, resulting in the non-randomness of the pores and measurement errors. The process parameters are laser power 190 W, scanning speed 700 mm/s, and the maximum average Vickers hardness value is 250.10 HV_0.2_. Under this process, the maximum Vickers hardness value of this test is 253.07 HV_0.2_. The process parameters are laser power 140 W, scanning speed 700 mm/s, and the minimum average Vickers hardness value is 218.73 HV_0.2_. In addition, the minimum Vickers hardness value of this test is 217.83 HV_0.2_ under this process. When the scanning speed is constant, with the increase in laser power, the Vickers hardness has a significant upward trend. The Vickers hardness and tensile strength are positively correlated.

**Figure 22:**
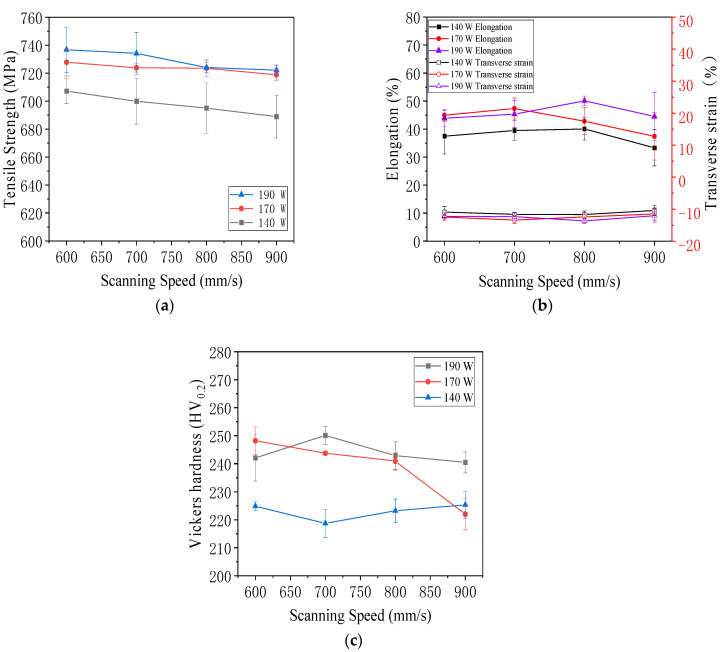
(**a**) Effect of scanning speed on ultimate tensile strength. (**b**) Effect of scanning velocity on elongation and lateral strain. (**c**) Effect of scanning speed on Vickers hardness.

As shown in Figure 22a, the scanning speed affects the ultimate tensile strength of the LPBF-built tensile specimen. The median value is the average of three groups of specimens, and the error is the standard deviation. It is easy to see from the data and images that when the laser power is constant, the ultimate tensile strength of the LPBF-built tensile specimen decreases with the scanning speed’s increase. Therefore, it can be concluded that the scanning speed is negatively correlated with the ultimate tensile strength of the LPBF-built tensile specimen.

As shown in Figure 22b, the effect of scanning speed on the elongation and transverse strain of LPBF tensile specimens was presented. The median value represented the average of three groups of specimens, and the error was the standard deviation. The elongation fluctuated between 50.11% and 33.33%, and the elongation of the experimental group with a laser power of 140 W was the lowest. The transverse strain showed no obvious change and remained stable at −10.42% to −13.67%, with minimal variation.

As shown in Figure 22c, the Vickers hardness of the experimental group with a laser power of 140 W is significantly the lowest, and the average hardness of the experimental groups with laser powers of 140 W and 190 W does not change significantly with the increase in scanning speed. When the laser power is 170 W, the Vickers hardness decreases significantly with the increase in scanning speed.

**Figure 23:**
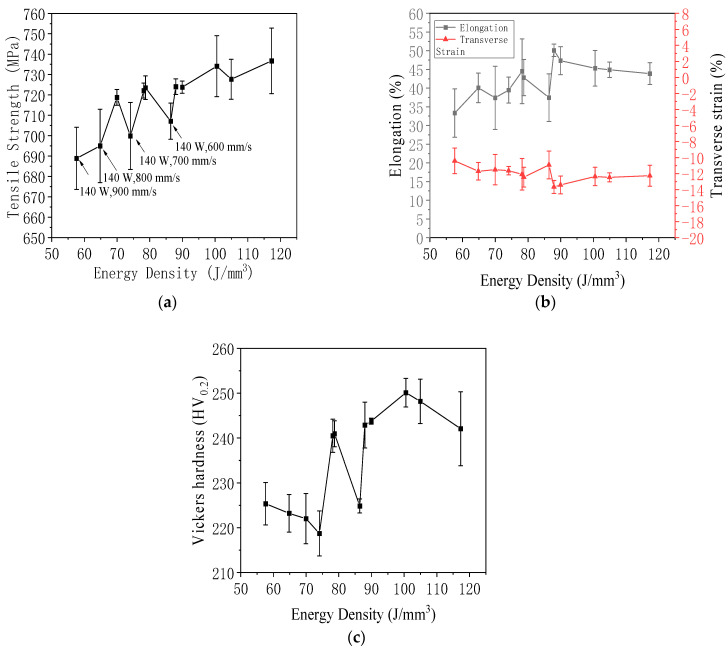
(**a**) Effect of energy density on tensile strength. (**b**) Effect of energy density on elongation. (**c**) Effect of energy density on Vickers hardness.

As shown in Figure 23a, as the energy density increases, the overall trend of tensile strength is to increase. The tensile strength increases with the increase in energy density, and energy density is used as an indicator to measure selective laser melting [61]. Compared with the experimental group with similar energy density, the tensile strength of the 140 W group is significantly lower. Therefore, this experiment found that energy density is not the only indicator determining tensile strength. When the energy density of two specimens is similar, the laser power plays a decisive role in their tensile strength.

Figure 23b illustrates the effect of energy density on the elongation and transverse strain of 316L stainless steel specimens fabricated by laser powder bed fusion. As energy density increased, elongation showed a general upward trend, indicating improved ductility. This enhancement was attributed to reduced porosity and stronger interlayer bonding at moderate to high energy inputs. At lower energy densities, elongation values were significantly lower, reflecting premature failure due to lack of fusion and the presence of internal defects. The transverse strain exhibited a corresponding pattern, with its absolute value increasing alongside elongation. This relationship reflected the Poisson effect typical of ductile metals, where materials with higher elongation also experienced greater lateral contraction. The inverse correlation between elongation and transverse strain was consistent with the behavior of 316L stainless steel, a material known to exhibit a positive Poisson’s ratio. These findings suggested that energy density played a critical role not only in determining strength but also in influencing the material’s plastic deformation characteristics. Optimizing energy input was therefore essential to balance strength and ductility for structural applications.

Figure 23c shows the relationship between energy density and Vickers hardness in 316L stainless steel specimens fabricated by laser powder bed fusion. As energy density increased, Vickers hardness exhibited a general upward trend. This was attributed to improved metallurgical bonding, reduced porosity, and more uniform microstructures achieved at higher energy input levels. The minimum average hardness of 218.37 HV_0.2_ was observed at 74.07 J/mm^3^ (140 W, 700 mm/s), while the maximum average value reached 250.10 HV_0.2_ at 100.53 J/mm^3^ (190 W, 700 mm/s). Although the energy density at 86.42 J/mm^3^ (140 W, 600 mm/s) was close to the optimal range, the hardness remained lower than expected (224.87 HV_0.2_), likely due to inconsistent melting or pore formation. The standard deviation in hardness was larger at both low and high extremes of energy density, suggesting increased variability due to defect concentration, such as a lack of fusion at low energy input and keyhole porosity or spatter at excessively high levels. This variability underscored the sensitivity of hardness to local structural inhomogeneities. The observed trend indicated that an energy density window between 90 J/mm^3^ and 100 J/mm^3^ was optimal for achieving high and stable hardness in LPBF-fabricated 316L stainless steel.

In summary, the mechanical properties of LPBF-fabricated 316L stainless steel were strongly influenced by laser power, scanning speed, and resulting energy density. Optimal mechanical performance, including high tensile strength, elongation, and Vickers hardness, was achieved at moderate to high energy densities, particularly around 100 J/mm^3^. Insufficient energy input led to poor interlayer bonding and increased porosity, while excessively high input introduced variability through keyhole defects and spatter. The results demonstrated that the fine-tuning of process parameters is essential to balance strength, ductility, and hardness, ensuring reliable structural performance in additively manufactured components.

### 3.8. Fracture Analysis

**Figure 24:**
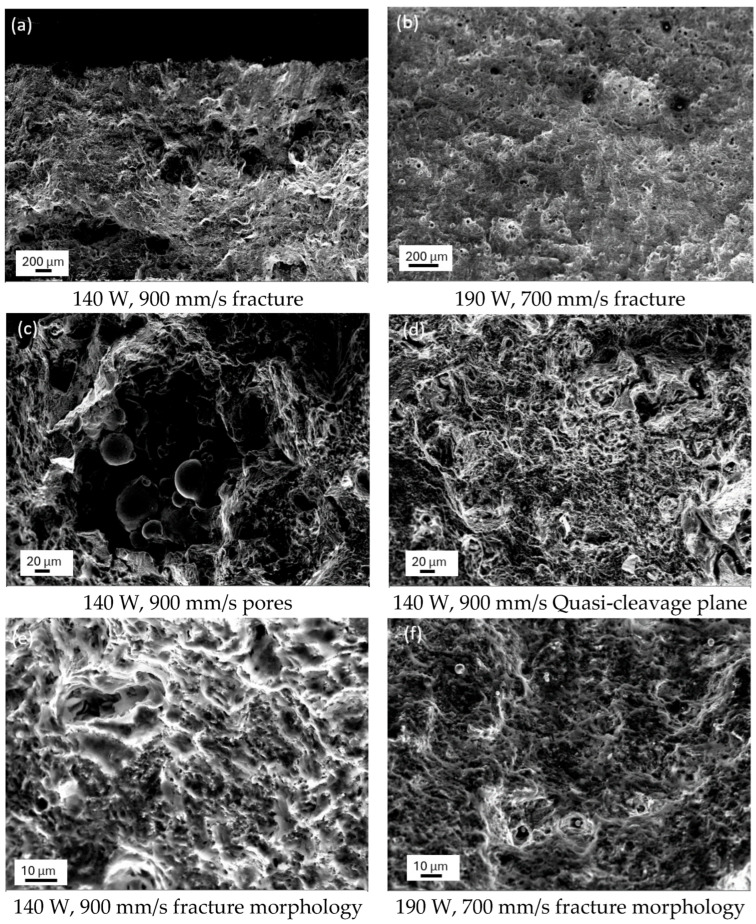
Fracture morphologies of 316L stainless steel specimens fabricated by laser powder bed fusion, comparing low-strength (**a**–**e**) and high-strength (**b**,**f**) conditions: (**a**) low-strength fracture surface with large pores; (**b**) high-strength fracture surface with minimal porosity; (**c**) enlarged pore with unmelted particles; (**d**) region near the pore showing quasi-cleavage features; (**e**) mixed-mode fracture with micropores and tearing ridges; (**f**) ductile fracture surface with fine dimples and partially melted particles.

Figure 24 presents the fracture morphologies of two 316L stainless steel specimens fabricated by laser powder bed fusion, one with the highest tensile strength and one with the lowest. As shown in Figure 24a, the low-strength specimen exhibited large pores with a measured porosity of 5.76%. In contrast, Figure 24b showed that the high-strength specimen contained only a few small pores, with a porosity of about 0.44%. Both fracture surfaces displayed numerous micro-pits known as dimples, which are indicative of ductile fracture. The average diameter of these dimples was 7 µm. These features were not pre-existing voids but were generated during plastic deformation, confirming a ductile fracture mechanism. Figure 24c showed an enlarged view of a pore from the low-strength sample, revealing numerous unmelted powder particles with diameters up to 109 µm. These particles acted as crack initiation sites during tensile loading. Figure 24d illustrates the region surrounding the pore, where short, dense tearing ridges and curved river-like patterns are visible. These features are characteristic of quasi-cleavage fracture, a transitional mode combining brittle cleavage and ductile tearing. Figure 24e further emphasizes this mixed fracture mode, showing both micropores and tearing edges. In contrast, Figure 24f reveals a fully dimpled fracture surface, representative of a ductile failure. Some dimples enclosed fine unmelted particles around 3.22 µm in diameter, indicating that even under high-energy input conditions, complete powder melting was not always achieved. These residual particles may reduce mechanical performance by serving as localized stress concentrators.

From a biomaterials perspective, the fracture behavior of LPBF-fabricated 316L stainless steel is of critical importance for the safe and effective function of load-bearing implants. Ductile fracture characterized by the presence of equiaxed dimples, as observed in low-porosity specimens, is generally desirable for biomedical applications because it indicates substantial energy absorption before failure and enhances the structural reliability of implants under physiological loading conditions [62]. This ductile mode of failure reduces the likelihood of sudden, catastrophic implant fracture, which is a key concern in orthopedic and dental applications. In contrast, the quasi-cleavage fracture surfaces found in specimens with higher porosity or unmelted particles may serve as early indicators of mechanical vulnerability. These features are often associated with brittle failure and lower energy absorption and may be linked to premature implant failure when subjected to cyclic loading in vivo [63].

The presence and distribution of defects not only lower the fatigue resistance of the material but can also affect the biological response, as crack initiation sites may create pathways for fluid ingress, promote localized corrosion, and facilitate the release of metal ions or particles into the surrounding tissue [64,65]. Such events have been associated with inflammation, impaired osseointegration, and ultimately reduced implant longevity [66]. Therefore, understanding and controlling fracture mechanisms through process parameter optimization are essential for developing LPBF 316L stainless steel implants that combine high mechanical performance with excellent biocompatibility and clinical safety.

## 4. Conclusions and Outlook

This study demonstrated that LPBF successfully produced 316L stainless steel components with excellent mechanical properties and fine grains, which are highly suitable for biomedical implant applications. By systematically varying laser power and scanning speed, we achieved nearly full-density 316L stainless steel with optimized tensile strength and Vickers hardness, both of which surpassed those of conventionally cast 316L. At the optimal process parameters—190 W laser power and 700 mm/s scanning speed—the fabricated specimens achieved a maximum tensile strength of 762.83 MPa, a Vickers hardness of 253.07 HV_0.2_, and a relative density of 99.97%. These properties indicated a superior ability to withstand physiological loads, which is essential for the long-term performance and reliability of orthopedic and dental implants.

A clear relationship was established between process parameters, microstructure, and mechanical properties. Careful control of energy density enabled the formation of both cellular and columnar austenitic grains, which formed under different cooling rates but within the same γ-Fe phase. The presence of fine cellular grains, resulting from rapid cooling, was associated with enhanced strength and hardness due to grain refinement, while the elongated columnar grains contributed to ductility and toughness. Importantly, the combination of these microstructural features offered a balance of mechanical performance that is highly desirable in biomaterials, where both high strength and adequate ductility are required to resist fracture and accommodate complex physiological stresses. Additionally, the microstructural homogeneity and continuity across the γ-Fe phase supported consistent biological responses, promoting favorable cell adhesion, proliferation, and integration with surrounding tissue.

We also found that minimizing internal defects, such as pores and unmelted powder, was essential for achieving high mechanical integrity and favorable biological outcomes. Specimens with porosity below 0.05% exhibited predominantly ductile fracture behavior, suggesting improved resistance to sudden failure—an important consideration for implants subject to cyclic loading in vivo. These high-density, low-porosity components are likely to support better tissue compatibility, reduce the risk of inflammation, and improve long-term implant survival.

Future research should integrate biological evaluation—such as cell compatibility studies and animal implantation experiments—to fully validate the clinical potential of LPBF-fabricated 316L stainless steel. The continued development of data-driven modeling and machine learning techniques may further accelerate the optimization of process parameters for tailored microstructure and properties. Finally, the unique ability of LPBF to precisely control microstructure and geometry paves the way for the next generation of patient-specific metallic biomaterials, addressing complex clinical needs with advanced performance and safety. Future work may expand the process window by exploring additional parameters such as hatch spacing and layer thickness to further optimize build quality. Detailed thermal modeling and in situ monitoring could help clarify the mechanisms behind grain morphology transitions. Investigating different build orientations and geometries would enhance understanding of anisotropy in practical components. Furthermore, extending the evaluation to include fatigue, creep, and high-temperature behavior would strengthen the applicability of LPBF parts in demanding environments. Advanced characterization techniques such as EBSD and X-ray tomography could offer deeper insight into grain structure and defect distribution, while studies on post-processing, including heat treatment and surface finishing, would aid in tailoring final properties for specific engineering applications.

In summary, this work clarified the critical process–structure–property relationships in LPBF-fabricated 316L stainless steel and demonstrated the considerable promise of this approach for functional biomaterials. By bridging the gap between engineering advances and biomedical requirements, LPBF 316L stainless steel offers a transformative solution for future orthopedic and dental implants.

## Figures and Tables

**Table 1:** Process parameters of 316L stainless steel tensile test specimens fabricated by LPBF.

P (W)	v (mm/s)	h (mm)	t (mm)	E (J/mm^3^)	Layer Rotation Angle
190	600	0.09	0.03	117.28	67°
190	700	0.09	0.03	100.53	67°
190	800	0.09	0.03	87.96	67°
190	900	0.09	0.03	78.19	67°
170	600	0.09	0.03	104.94	67°
170	700	0.09	0.03	89.95	67°
170	800	0.09	0.03	78.70	67°
170	900	0.09	0.03	69.96	67°
140	600	0.09	0.03	86.42	67°
140	700	0.09	0.03	74.07	67°
140	800	0.09	0.03	64.81	67°
140	900	0.09	0.03	57.61	67°

**Table 2:** Chemical composition of 316L stainless steel powder.

Element	Fe	Cr	Ni	Mo	Mn	Si	C	P	S	O
Content (wt%)	Bal.	16~18	10~14	2.0~3.0	≤2.0	≤1.0	≤0.03	≤0.0045	≤0.03	≤0.05

**Table 3:** EDS result of 316L specimens fabricated by LPBF.

Area			Elemental Concentration (wt%)						
	Fe	Ni	Cr	O	C	Si	Mo	S	Mn
Map 1	50.32	26.64	13.73	4.42	1.85	0.90	2.12	—	0.02
Map 2	44.90	31.98	11.96	5.22	1.65	1.05	2.01	1.18	0.05
Line 1	94.33	—	—	—	5.67	—	—	—	—
Line 2	79.34	—	17.45	—	3.21	—	—	—	—

**Table 4:** Mechanical properties dataset [58] of 316L specimens fabricated by LPBF.

Laser Power (W)	Scanning Speed (mm/s)	Energy Density (J/mm^3^)	Tensile Strength (MPa)	Vickers Hardness (HV_0.2_)
190	600	117.28	736.72 ± 16.08	242.06 ± 8.22
190	700	100.53	734.10 ± 14.93	250.10 ± 3.20
190	800	87.96	724.01 ± 3.83	242.87 ± 5.12
190	900	78.189	722.10 ± 3.68	240.47 ± 3.71
170	600	104.94	727.70 ± 9.81	248.17 ± 4.96
170	700	89.95	723.81 ± 3.02	243.74 ± 0.67
170	800	78.7	723.53 ± 5.81	240.93 ± 2.89
170	900	69.96	718.80 ± 3.84	222.03 ± 5.61
140	600	86.42	707.10 ± 8.89	224.87 ± 1.58
140	700	74.07	699.88 ± 16.41	218.73 ± 5.02
140	800	64.81	695.00 ± 18.00	223.23 ± 4.19
140	900	57.61	688.88 ± 15.27	225.36 ± 4.74

## Data Availability

The original contributions presented in the study are included in the article/supplementary material, further inquiries can be directed to the corresponding author.

## Supplementary Materials

The following supporting information can be downloaded at: https://www.mdpi.com/article/10.3390/jfb16080280/s1, Figure S1. Farsoon FS121M Laser powder bed fusion Equipment. Figure S2. Laser spot calibration. Figure S3. Digital image correlation (DIC) strain contour maps of 316L stainless steel tensile specimens fabricated by LPBF: (a) transverse strain in the elastic stage, (b) longitudinal strain in the elastic stage, (c) transverse strain in the strengthening stage, (d) longitudinal strain in the strengthening stage, (e) transverse strain during necking, (f) longitudinal strain during necking, (g) transverse strain at fracture, and (h) longitudinal strain at fracture. Figure S4. SEM and energy-dispersive X-ray spectroscopy (EDS) mapping of LPBF-fabricated 316L stainless steel: (a) SEM secondary electron image, (b) SEM backscattered electron image, (c) Fe elemental map, (d) Ni elemental map, (e) Mo elemental map, (f) Cr elemental map. Figure S5. Tensile specimens and speckled tensile specimens. Figure S6. Fractured 316L specimens after tensile test. Figure S7. Cross-sectional metallographic images of 316L stainless steel specimens fabricated by LPBF with an interlayer rotation angle of 67°, showing molten pool morphology under varying laser powers and scanning speeds: (a–d) 190 W, (e–h) 170 W, and (i–l) 140 W at scanning speeds of 600 mm/s, 700 mm/s, 800 mm/s, and 900 mm/s, respectively. Figure S8. BD-TD metallographic diagram of specimens with interlaminar rotation angle of 0 degree. Figure S9.. BD-TD metallographic diagram of specimens with interlaminar rotation angle of 0 degree. Figure S10. Vertical metallographic diagram of specimens with interlayer rotation angle of 0 degree. Figure S11. Parallel metallographic diagram of specimens with interlayer rotation angle of 0 degree.
