# Supplementary material for: Manufacturing, Microstructure, and Mechanics of 316L SS Biomaterials by Laser Powder Bed Fusion
Source: J Funct Biomater. 2025 Jul 31;16(8):280. doi: 10.3390/jfb16080280 (PMC12387571; doi:10.3390/jfb16080280)
Supplement: Supplementary file 1 [file jfb-16-00280-s001.zip › jfb-3747731-supplementary.pdf]

Supplementary Information

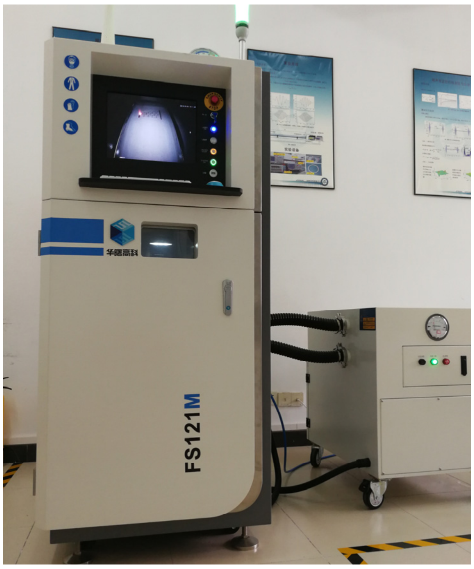

Figure S1. Farsoon FS121M Laser powder bed fusion Equipment.

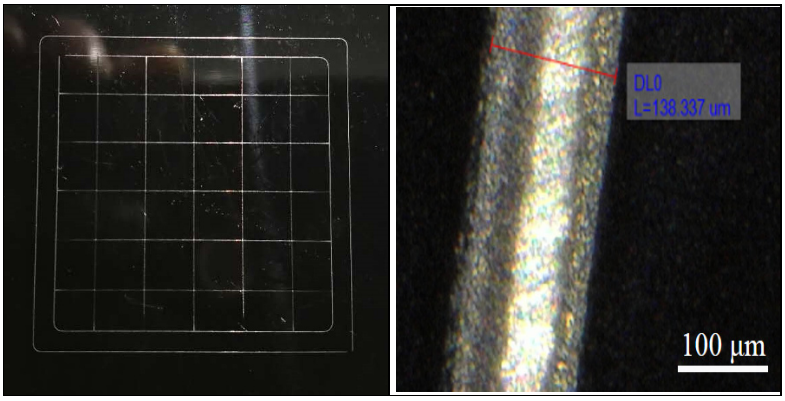

Figure S2. Laser spot calibration.

Digital Image Correlation (DIC) experimental steps:

(1) First, the specimens were numbered and the gauge length was marked. In this experiment, the gauge length was calculated as 31 mm according to the GB/T 228.1-2010 standard. The cross-sectional area was calculated based on the thickness and width of the test piece. Finally, the paint was sprayed to mark the points and the gauge length.

(2) A high-speed camera was set up, and the acquisition computer was connected to the camera via a data cable. The exposure light, the position of the camera, the aperture and the focal length were adjusted so that the lens was facing the specimen and a clear picture without reflection was obtained.

(3) The image acquisition software Vic-Snap was opened for calibration and photo acquisition. The photo acquisition time in this experiment was 500 ms.

(4) After the experiment, strain analysis was performed on the acquired photos to derive the transverse and longitudinal strain and elongation data of the specimen.

The definitions of longitudinal and transverse strain in the DIC analysis were based on the geometry and loading axis of the tensile specimen itself. The longitudinal direction corresponded to the axis of applied tensile force, while the transverse direction was perpendicular

to it, regardless of the build direction or the laser scanning direction during LPBF fabrication. All DIC strain contour maps therefore reflected deformation relative to the specimen coordinates.

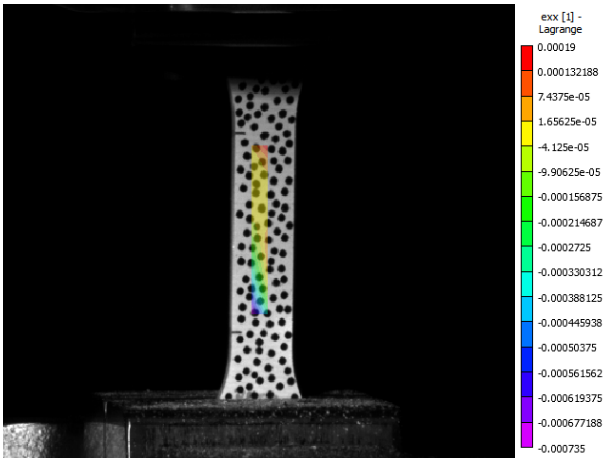

(a) Transverse strain contour diagram in the elastic stage

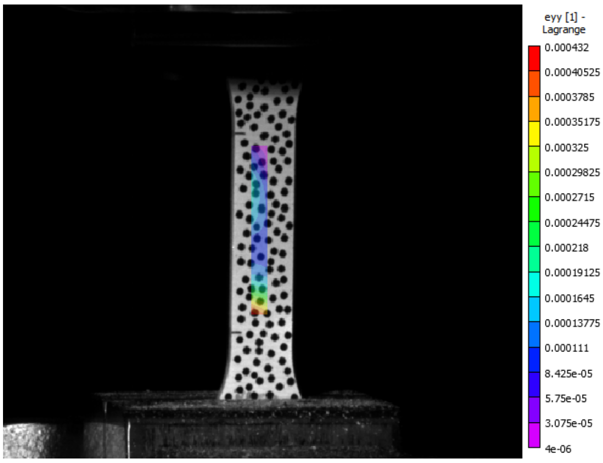

(b) Longitudinal strain contour diagram in the elastic stage

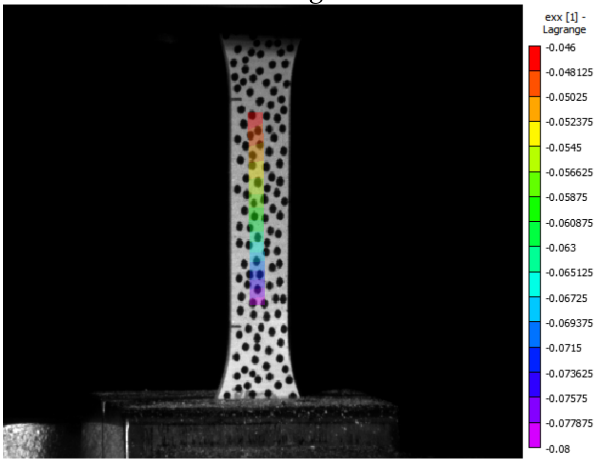

(c) Transverse strain contour diagram during the strengthening stage

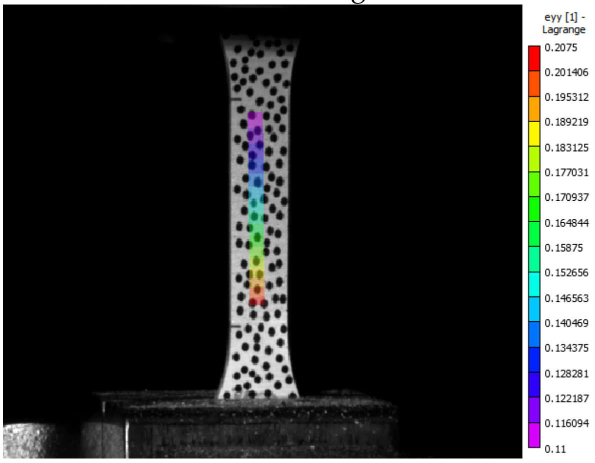

(d) Longitudinal strain contour diagram during the strengthening stage

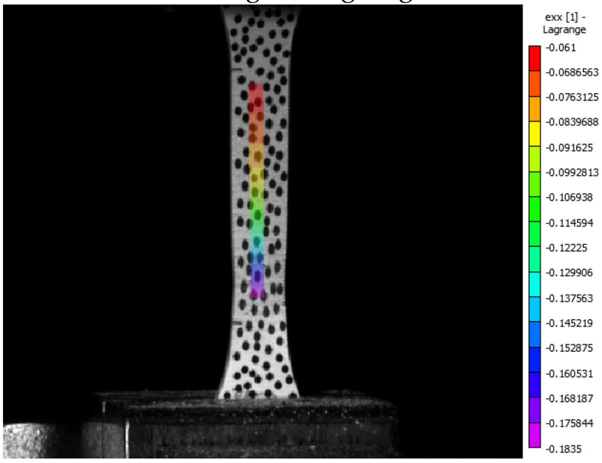

(e) Transverse strain contour diagram during the necking stage

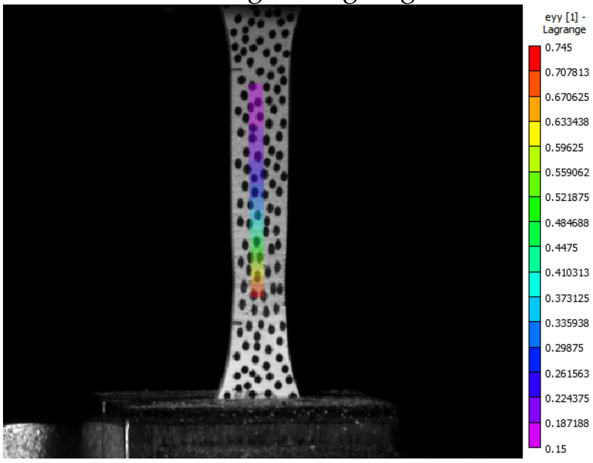

(f) Longitudinal strain contour diagram during the necking stage

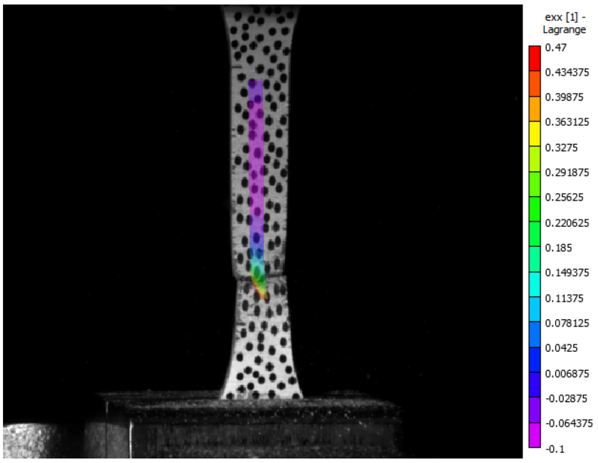

(g) Transverse strain cloud diagram at the moment of fracture

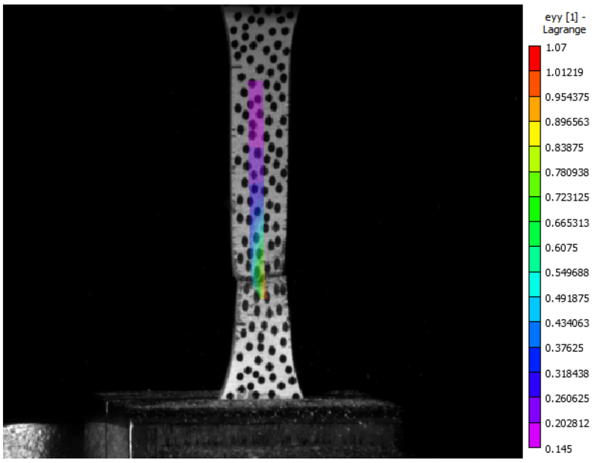

(h) Longitudinal strain cloud diagram at the moment of fracture

Figure S3. Digital image correlation (DIC) strain contour maps of 316L stainless steel tensile specimens fabricated by LPBF: (a) transverse strain in the elastic stage, (b) longitudinal strain in the elastic stage, (c) transverse strain in the strengthening stage, (d) longitudinal strain in the strengthening stage, (e) transverse strain during necking, (f) longitudinal strain during necking, (g) transverse strain at fracture, and (h) longitudinal strain at fracture.

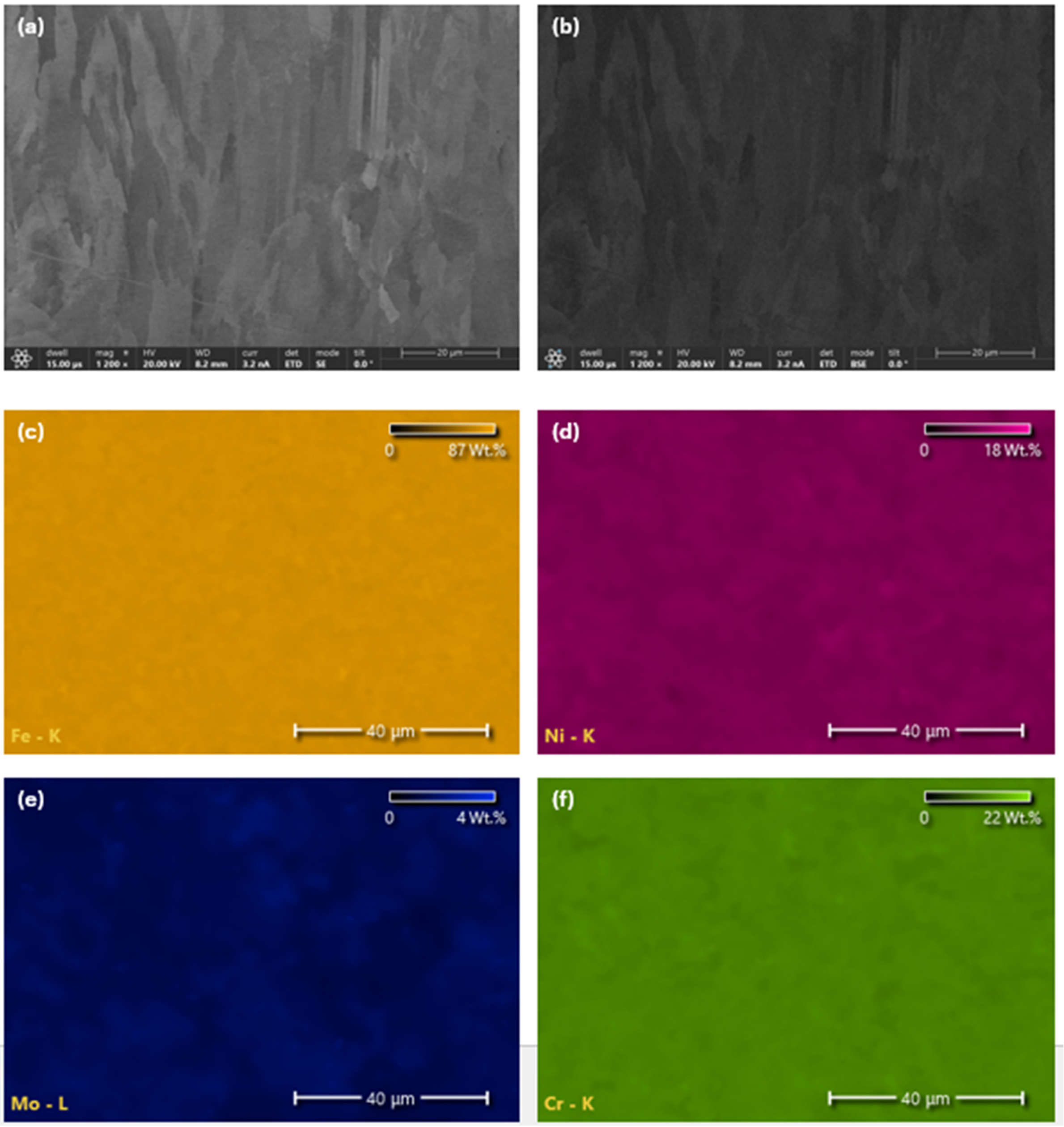

Figure S4. SEM and energy-dispersive X-ray spectroscopy (EDS) mapping of LPBF-fabricated 316L stainless steel: (a) SEM secondary electron image, (b) SEM backscattered electron image, (c) Fe elemental map, (d) Ni elemental map, (e) Mo elemental map, (f) Cr elemental map.

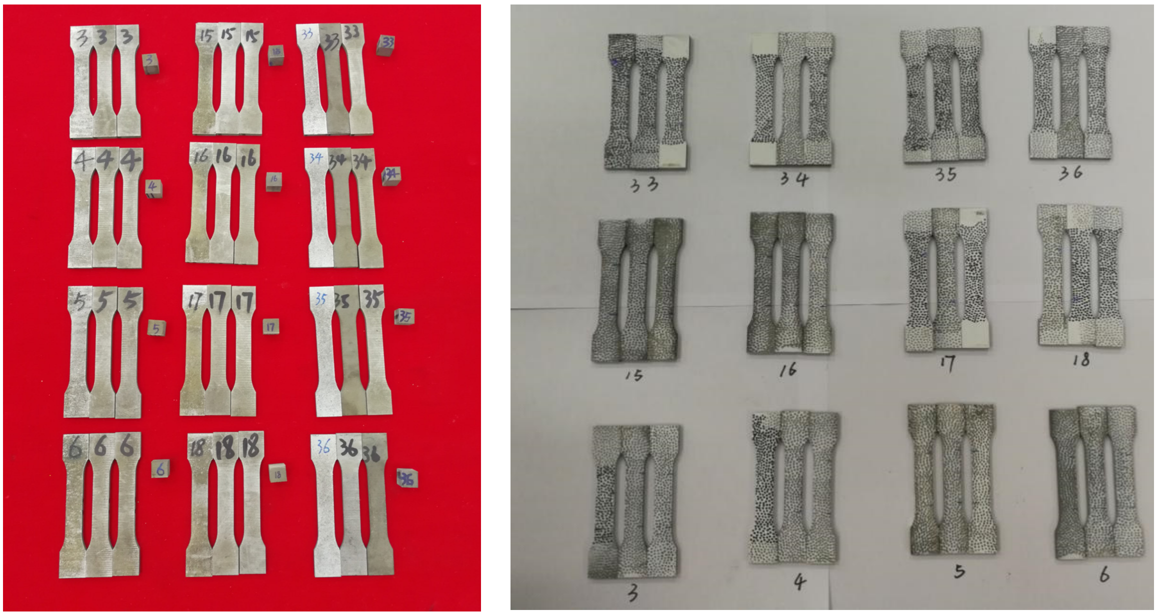

Figure S5.Tensile specimens and speckled tensile specimens.

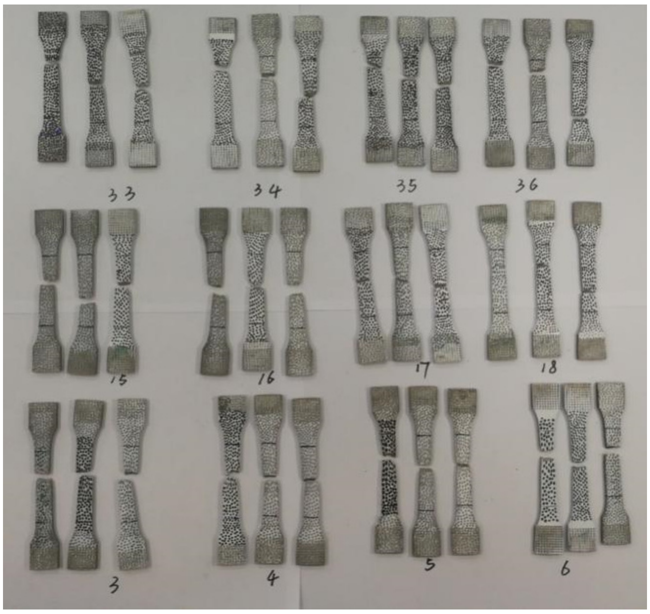

Figure S6. Fractured 316L specimens after tensile test.

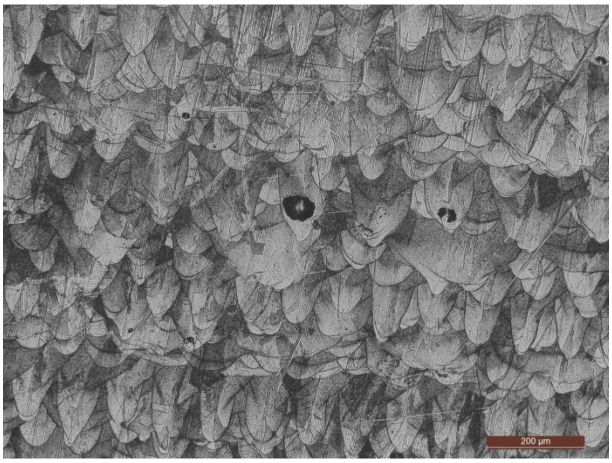

(a) 190 W，600 mm/s

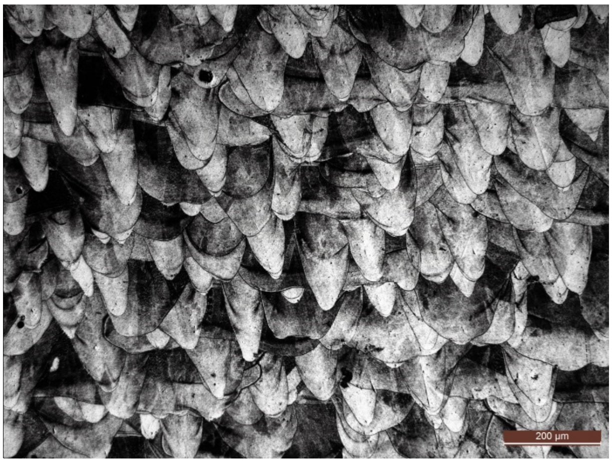

(b) 190 W，700 mm/s

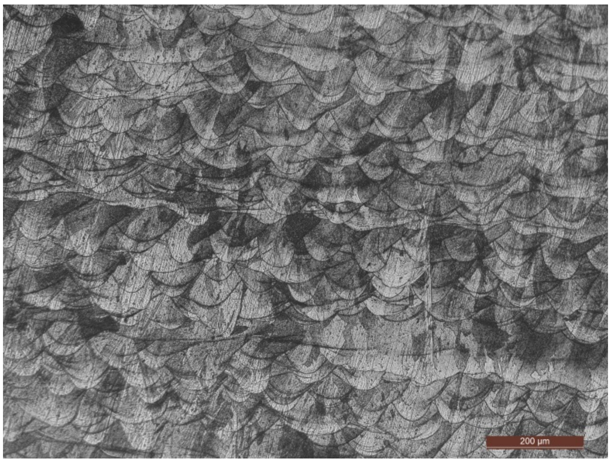

(c) 190 W，800 mm/s

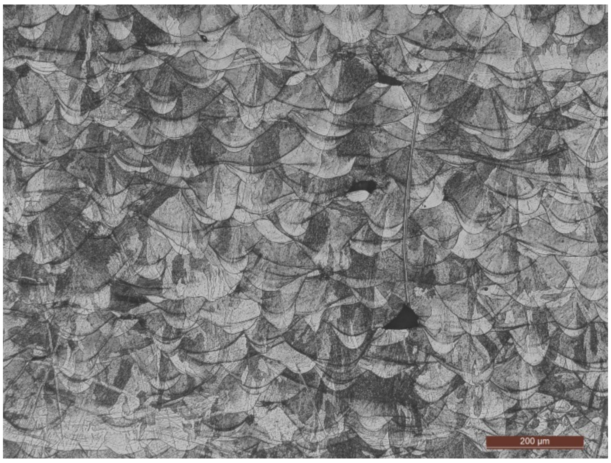

(d) 190 W，900 mm/s

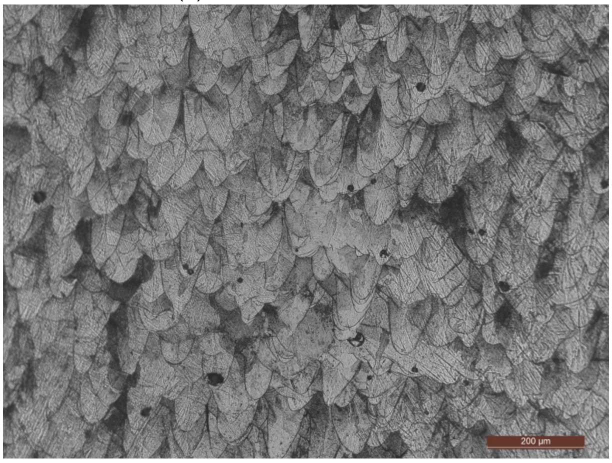

(e) 170 W，600 mm/s

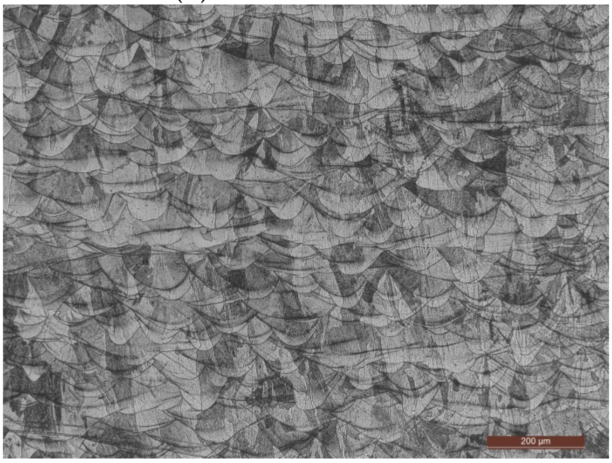

(f) 170 W，700 mm/s

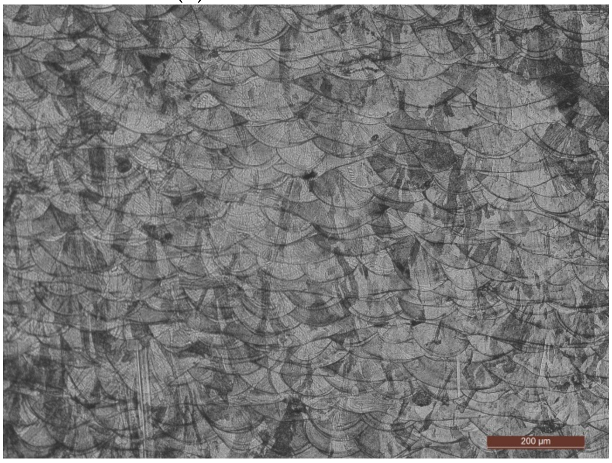

(g) 170 W，800 mm/s

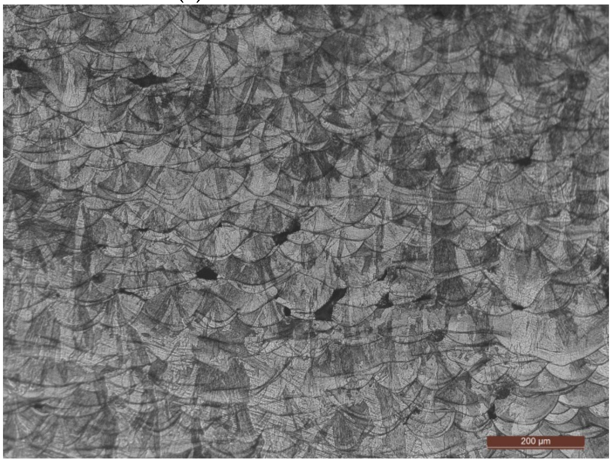

(h) 170 W，900 mm/s

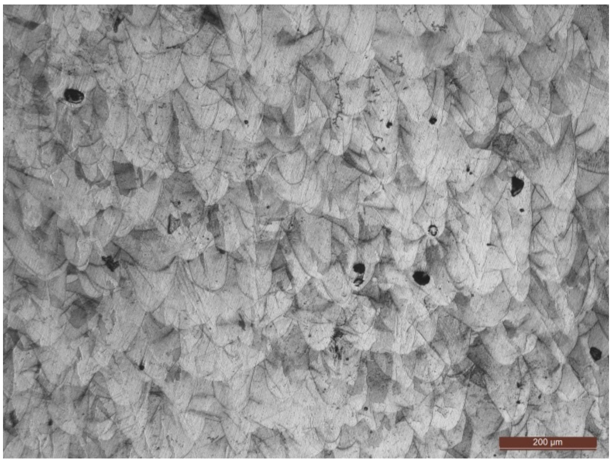

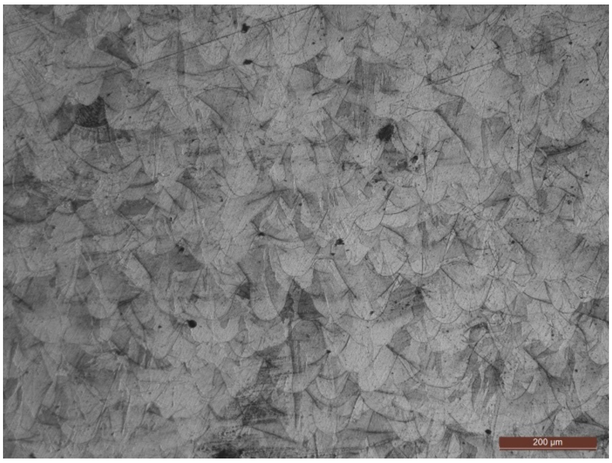

(i) 140 W，600 mm/s　　(j) 140 W，700 mm/s

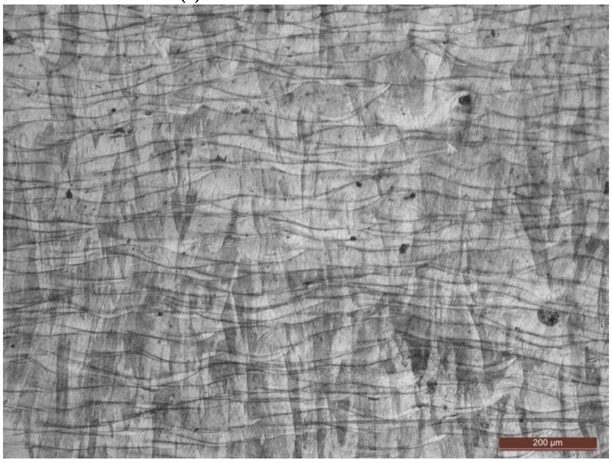

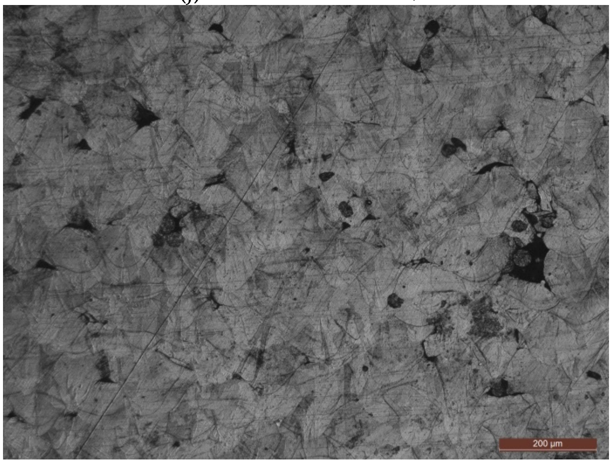

(k) 140 W，800 mm/s　　(l) 140 W，900 mm/s

Figure S7. Cross-sectional metallographic images of 316L stainless steel specimens fabricated by LPBF with an interlayer rotation angle of 67°, showing molten pool morphology under varying laser powers and scanning speeds: (a–d) 190 W, (e–h) 170 W, and (i–l) 140 W at scanning speeds of 600 mm/s, 700 mm/s, 800 mm/s, and 900 mm/s, respectively.

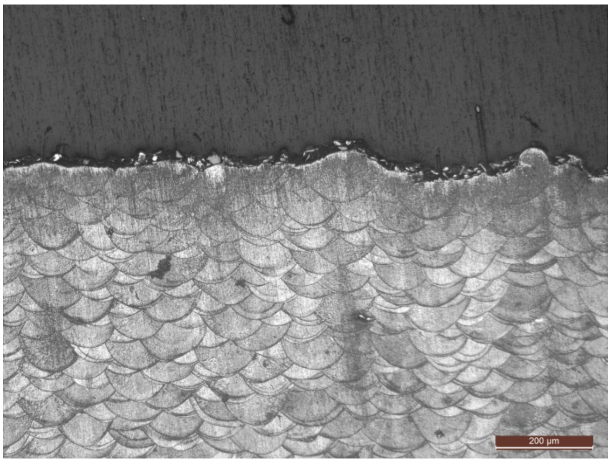

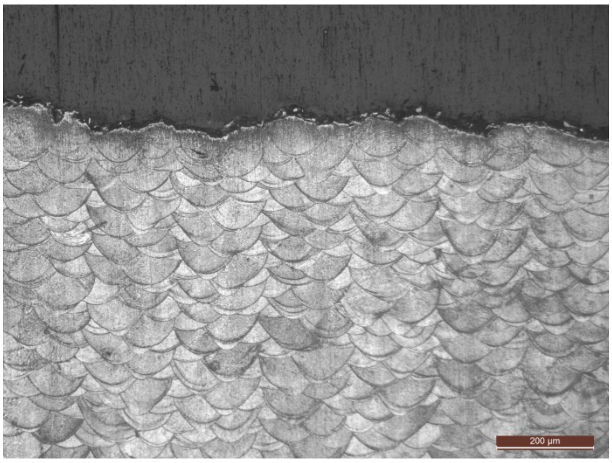

(a) 140 W，600 mm/s　　(b) 140 W，700 mm/s

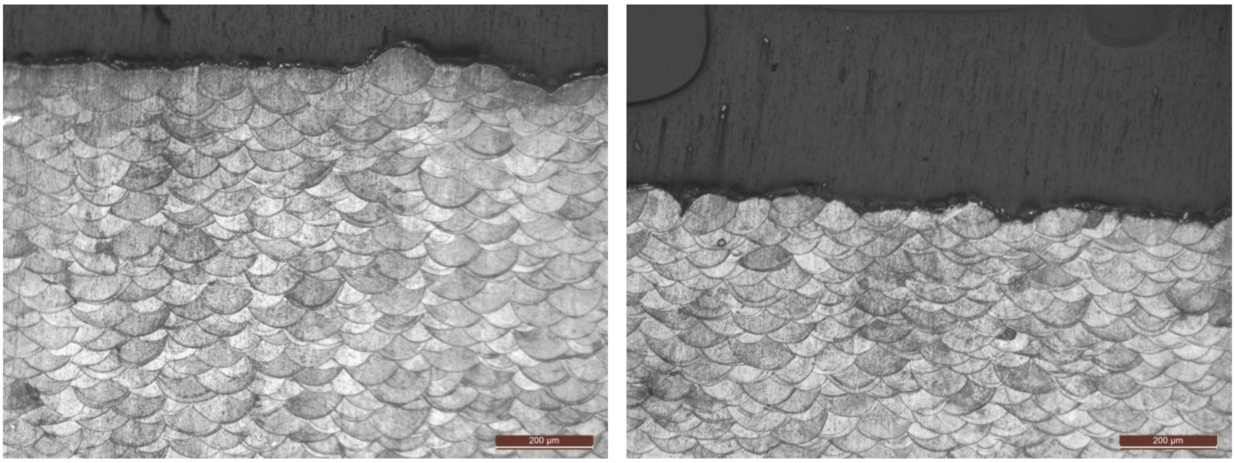

(c) 140 W，800 mm/s (d) 140 W，900 mm/s

Figure S8. BD-TD metallographic diagram of specimens with interlaminar rotation angle of 0 degree.

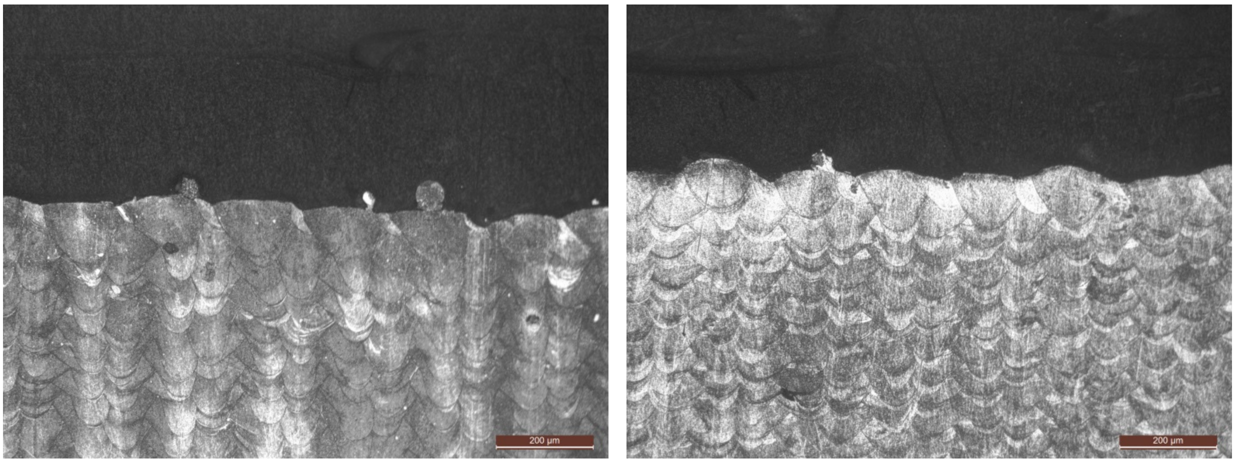

(a) 170 W，600 mm/s (b) 170 W，700 mm/s

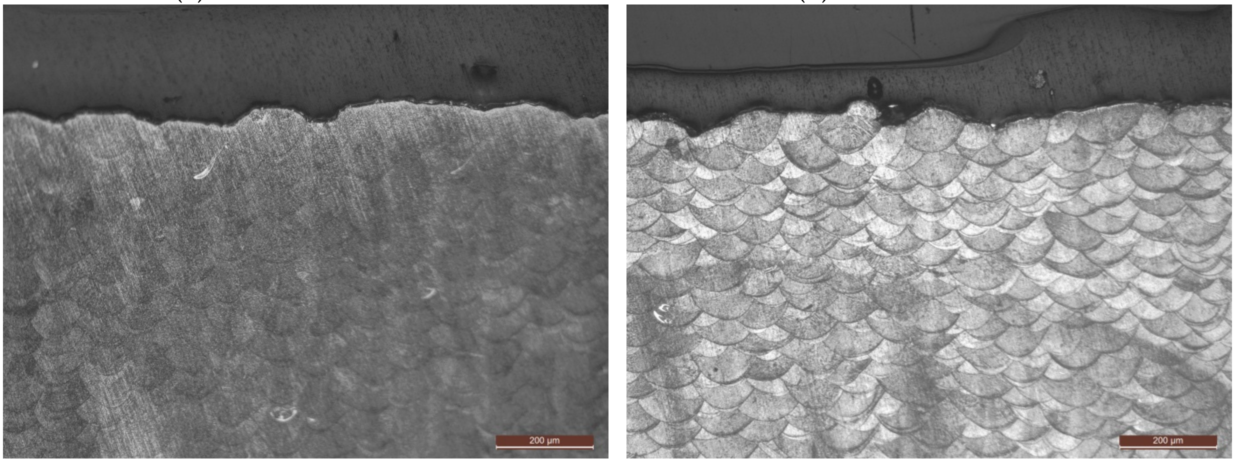

(c) 170 W，800 mm/s (d) 170 W，900 mm/s

Figure S9. BD-TD metallographic diagram of specimens with interlaminar rotation angle of 0 degree.

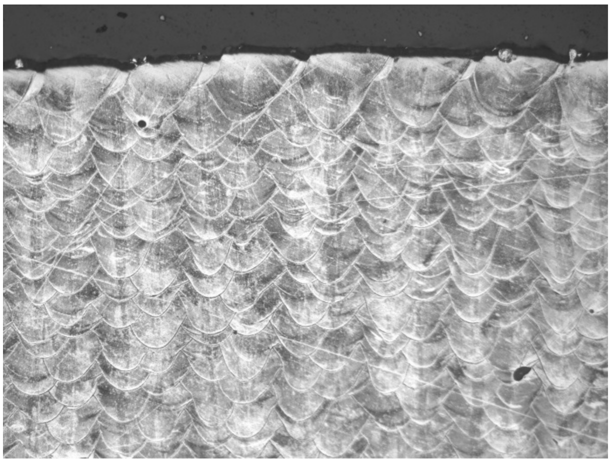

(a) 190 W，600 mm/s

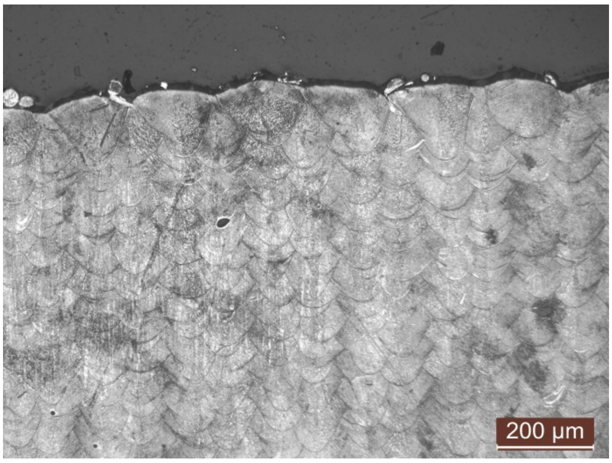

(b) 190 W，700 mm/s

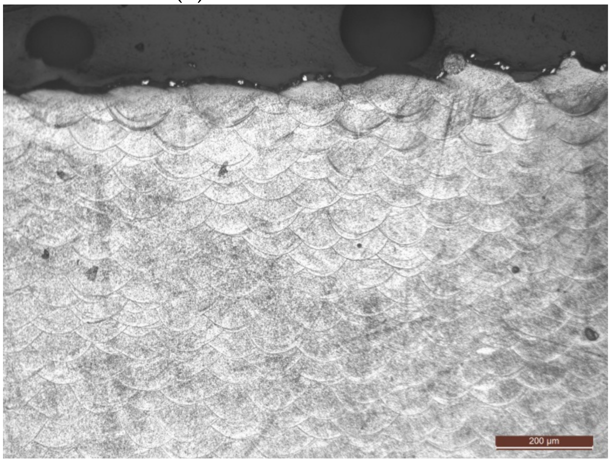

(c) 190 W，800 mm/s

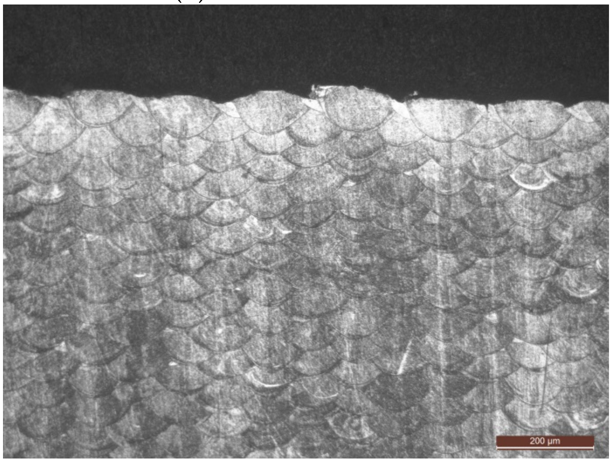

(d) 190 W，900 mm/s

Figure S10. Vertical metallographic diagram of specimens with interlayer rotation angle of 0 degree.

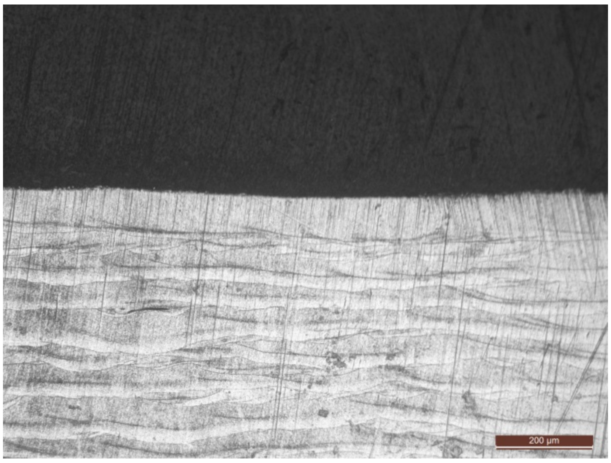

(a) 190 W，600 mm/s

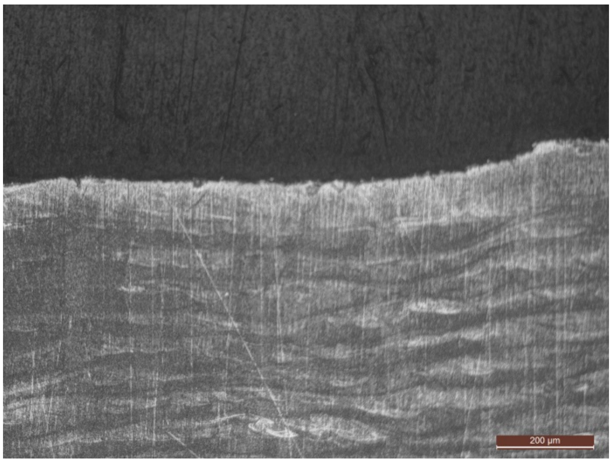

(b) 190 W，700 mm/s

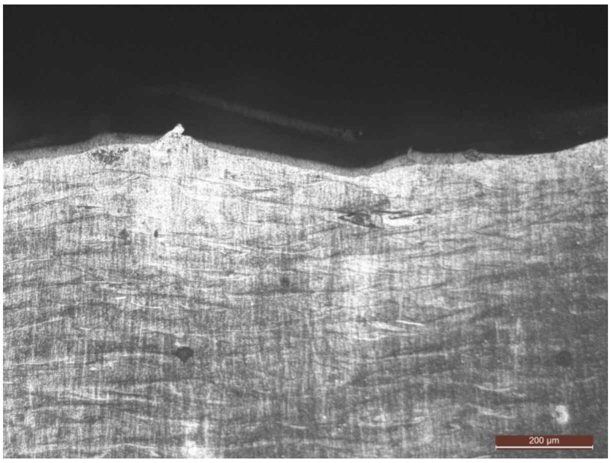

(c) 190 W，800 mm/s

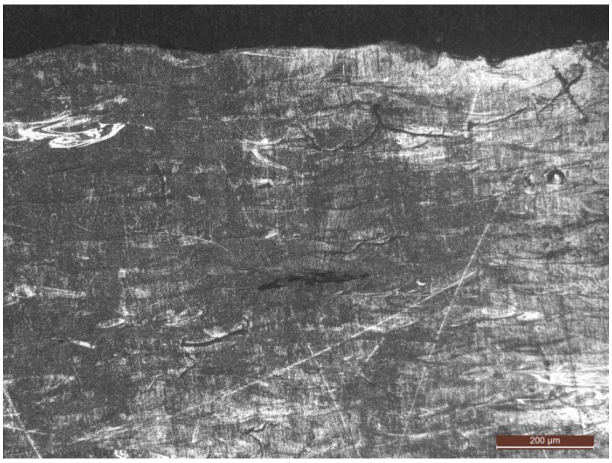

(d) 190 W，900 mm/s

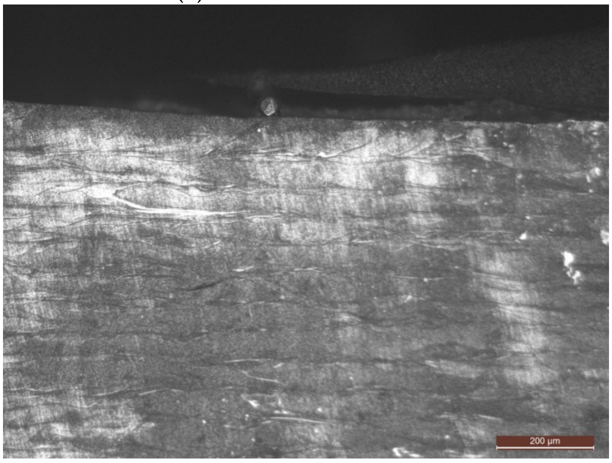

(e) 170 W，600 mm/s

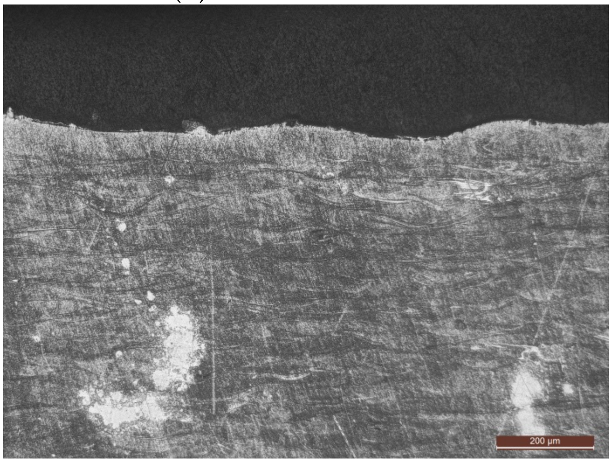

(f) 170 W，700 mm/s

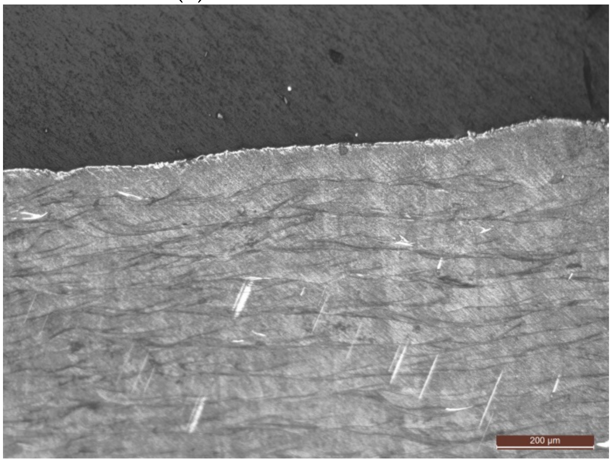

(g) 170 W，800 mm/s

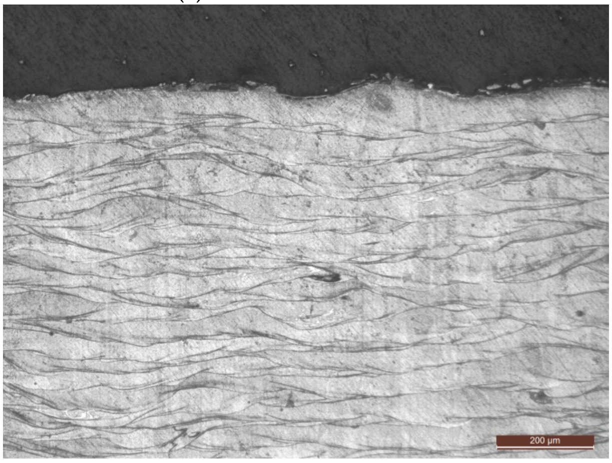

(h) 170 W，900 mm/s

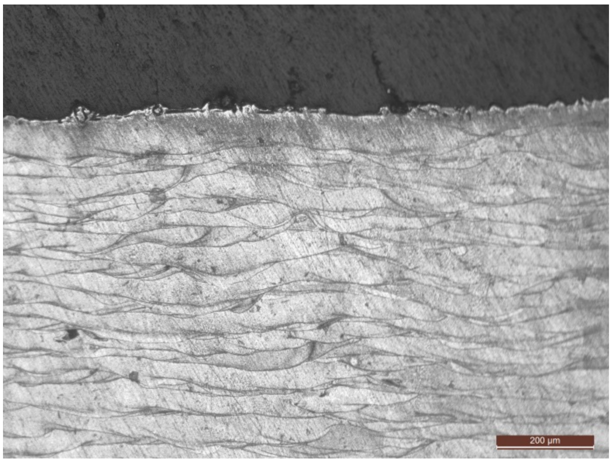

(i) 140 W，600 mm/s

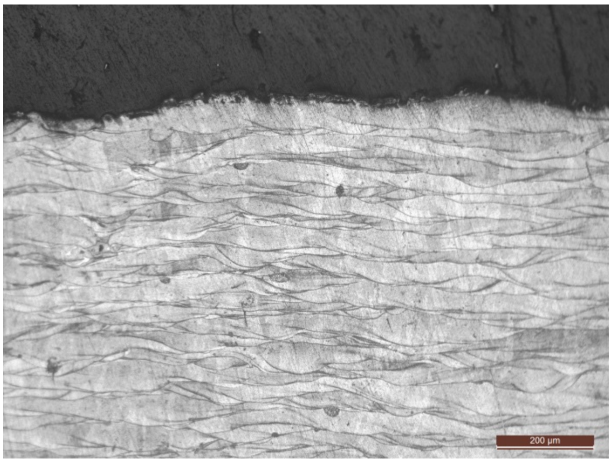

(j) 140 W，700 mm/s

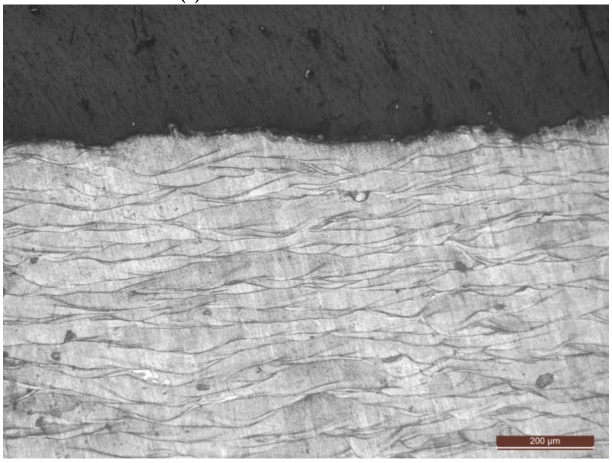

(k) 140 W，800 mm/s

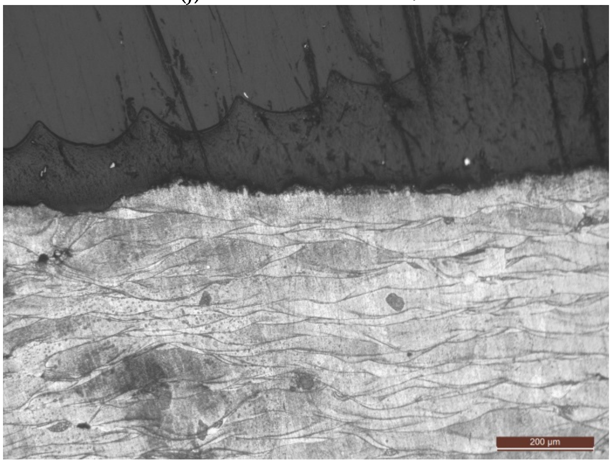

(l) 140 W，900 mm/s

Figure S11. Parallel metallographic diagram of specimens with interlayer rotation angle of 0 degree.
